# Persistent luminescence nanoparticles for cancer theranostics application

**DOI:** 10.1186/s12951-021-00862-z

**Published:** 2021-04-20

**Authors:** Nian Liu, Xiao Chen, Xia Sun, Xiaolian Sun, Junpeng Shi

**Affiliations:** 1 Xiamen Cardiovascular Hospital, Xiamen University, Xiamen, 361015 China; 2 State Key Laboratory of Natural Medicines, Key Laboratory of Drug Quality Control and Pharmacovigilance, Department of Pharmaceutical Analysis, China Pharmaceutical University, Nanjing, 210009 China; 3 Department of Chemistry, Technical University of Munich, 85747 Garching, Germany; 4 Medizinische Klinik Und Poliklinik IV, Ludwig-Maximilians-Universität München, 80336 Munich, Germany; 5 Key Laboratory of Design and Assembly of Functional Nanostructures, Fujian Institute of Research On the Structure of Matter, Chinese Academy of Sciences, Fuzhou, 350002 China; 6 Department of Translational Medicine, Xiamen Institute of Rare Earth Materials, Chinese Academy of Sciences, Xiamen, 361021 China

**Keywords:** Persistent luminescence nanoparticles, Synthesis, Surface modification, PersL imaging, Multiple excitation sources, Theranostics

## Abstract

Persistent luminescence nanoparticles (PLNPs) are unique optical materials that emit afterglow luminescence after ceasing excitation. They exhibit unexpected advantages for in vivo optical imaging of tumors, such as autofluorescence-free, high sensitivity, high penetration depth, and multiple excitation sources (UV light, LED, NIR laser, X-ray, and radiopharmaceuticals). Besides, by incorporating other functional molecules, such as photosensitizers, photothermal agents, or therapeutic drugs, PLNPs are also widely used in persistent luminescence (PersL) imaging-guided tumor therapy. In this review, we first summarize the recent developments in the synthesis and surface functionalization of PLNPs, as well as their toxicity studies. We then discuss the in vivo PersL imaging and multimodal imaging from different excitation sources. Furthermore, we highlight PLNPs-based cancer theranostics applications, such as fluorescence-guided surgery, photothermal therapy, photodynamic therapy, drug/gene delivery and combined therapy. Finally, future prospects and challenges of PLNPs in the research of translational medicine are also discussed.

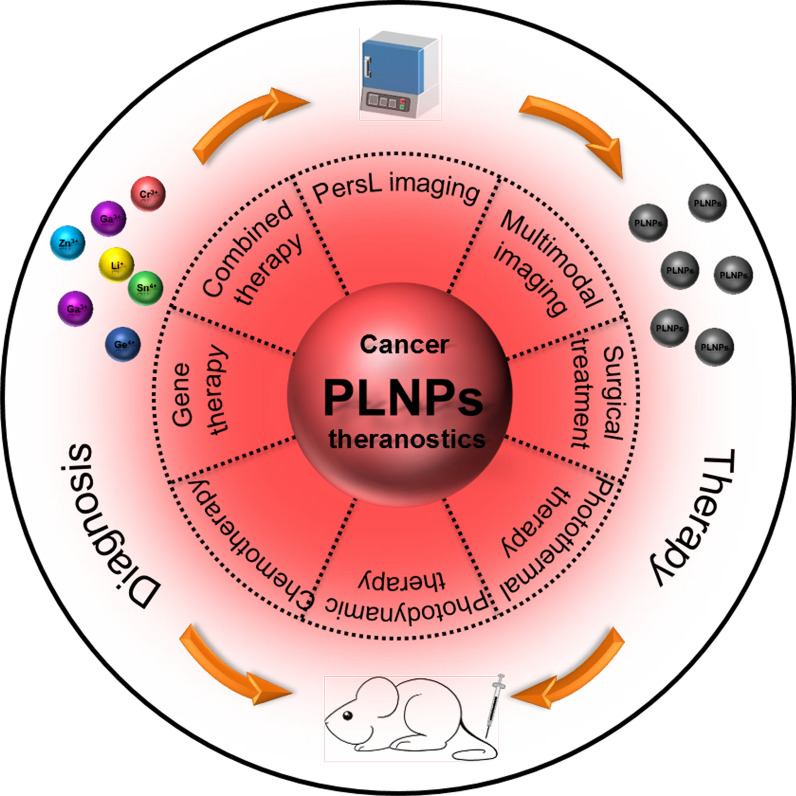

## Introduction

With the increasing number incidence of fatal diseases like cancer, there is a growing need for precise diagnosis and efficient therapy [1]. Therefore, nanomedicines have been proposed to use nanotechnology to endow both imaging and therapeutic capabilities to achieve cancer theranostics [2–6]. Although a range of nanomaterials have been developed for biomedical applications, persistent luminescence nanoparticles (PLNPs) as unique optical materials have attracted extensive attention for excitation-free optical imaging and cancer therapy due to persistent luminescence (PersL) property and nanocarrier structures [7, 8]. PLNPs can store part of the excitation energy and then release the photonic emission for an appreciable time after ceasing excitation. Such continuous luminescence phenomenon is called PersL or afterglow luminescence, which is achieved by forming defects by doping emitter ions in a specific host, and capturing and releasing electrons through these defects [9, 10]. By tuning the host and emitter, it is possible to obtain PLNPs with different emission wavelengths from UV to near-infrared (NIR) region [9].

In recent years, PLNPs exhibit outstanding strengths in the field of tumor diagnosis and treatment. Firstly, compared to traditional fluorescent agents (such as semiconductor quantum dots [11, 12], upconversion nanoparticles [13, 14], organic dyes [15, 16]), optical imaging with PLNPs can be freed from real-time excitation by external light sources, thus completely avoiding interference of autofluorescence and enabling high-sensitive in vivo imaging. Secondly, PLNPs can be excited by multiple excitation sources, such as UV, LED, NIR laser, X-ray, and radiopharmaceuticals, which overcome the poor imaging quality and the poor penetration depth caused by short-wavelength excitation [17]. Thirdly, PLNPs can be easily doped or modified with elements/ligands from other imaging modalities to enable multimodal imaging of live subjects, which provide more sensitive and accurate information for disease diagnostics. Fourth, PLNPs with hollow or mesoporous structures are also suited for drug delivery. Owing to their versatile surface functionality, photothermal agents, chemodrugs, photosensitizers (PSs) or genes can be easily loaded into the PLNPs nanoplatforms for PersL imaging-guided therapy. Inspired by these characteristic advantages of PLNPs, an increasing number of researches have been reported for PLNPs-based bioimaging and therapy. Though some reviews have been shown from different aspects of PLNPs field [7, 9, 17–21], it is still important to make a new summarization to stress on the cancer theranostics application using the rational designed PLNPs nanoplatforms.

**Fig. 1:**
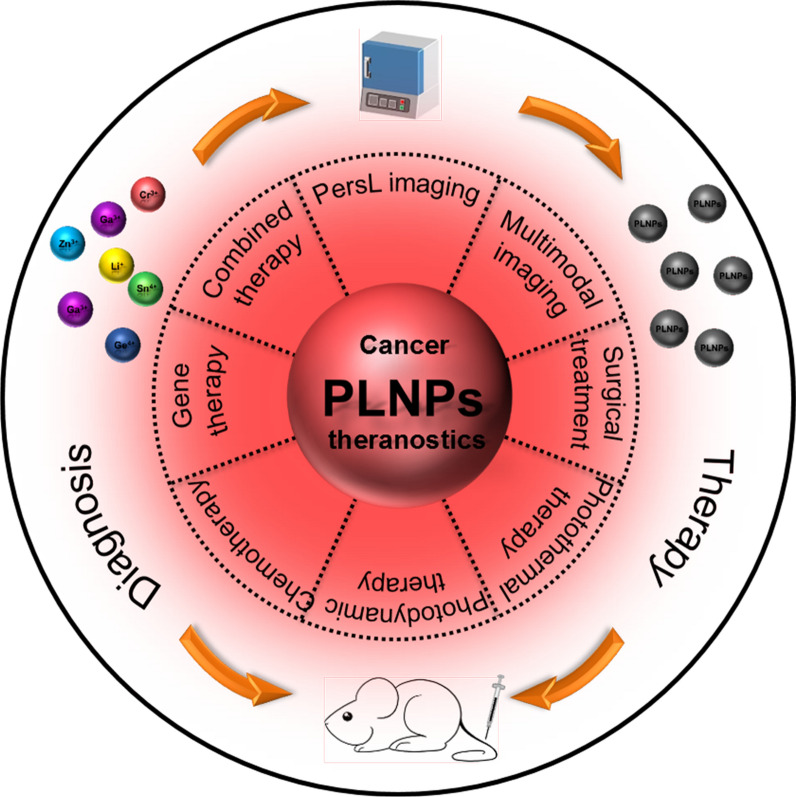
Graphic illustration of the cancer theranostics application of PLNPs

 Herein, we primarily summarize the recent progress of biomedical PLNPs from the rational design of PLNPs nanoplatforms to the cancer theranostics application (Fig. 1). Instead of giving a complete historical report of PLNPs, we highlight the newly developed strategies for their synthesis methods, surface functionalization, and biosafety. Subsequently, we discuss the in vivo PersL imaging and multimodal imaging with different excitation sources. Then, we showcase the tumor theranostic applications of PLNPs, including PersL imaging-guided surgery, photothermal therapy (PTT), photodynamic therapy (PDT), chemotherapy, gene therapy, and combined therapy. Finally, we provide the future outlook for PLNPs with the challenging areas.

## PLNPs design considerations

### PersL mechanisms

Understanding the PersL mechanism of PLNPs does good for the rational design of PLNPs with long PersL and strong brightness. There are three basic elements in PLNPs: host, emitter, and traps. The host is the main body of PLNPs and acts as the carrier of the emitters. The composition and structure of the host have a certain influence on the spectral structure of the emitter, such as the shape and emission spectrum [9]. The emitters are usually served by rare-earth ions, transition metal ions, or main group elements in PLNPs, such as Eu^2+^, Sm^3+^, Cr^3+^, Mn^2+^, Bi^3+^, etc. The luminescent wavelength of PLNPs is mainly determined by their emitters [10, 22]. The traps are an energy state that can trap electrons in the forbidden band. The traps are usually formed by intrinsic defects or ion doping into the host, which determines the PL time and intensity [23, 24].

**Fig. 2:**
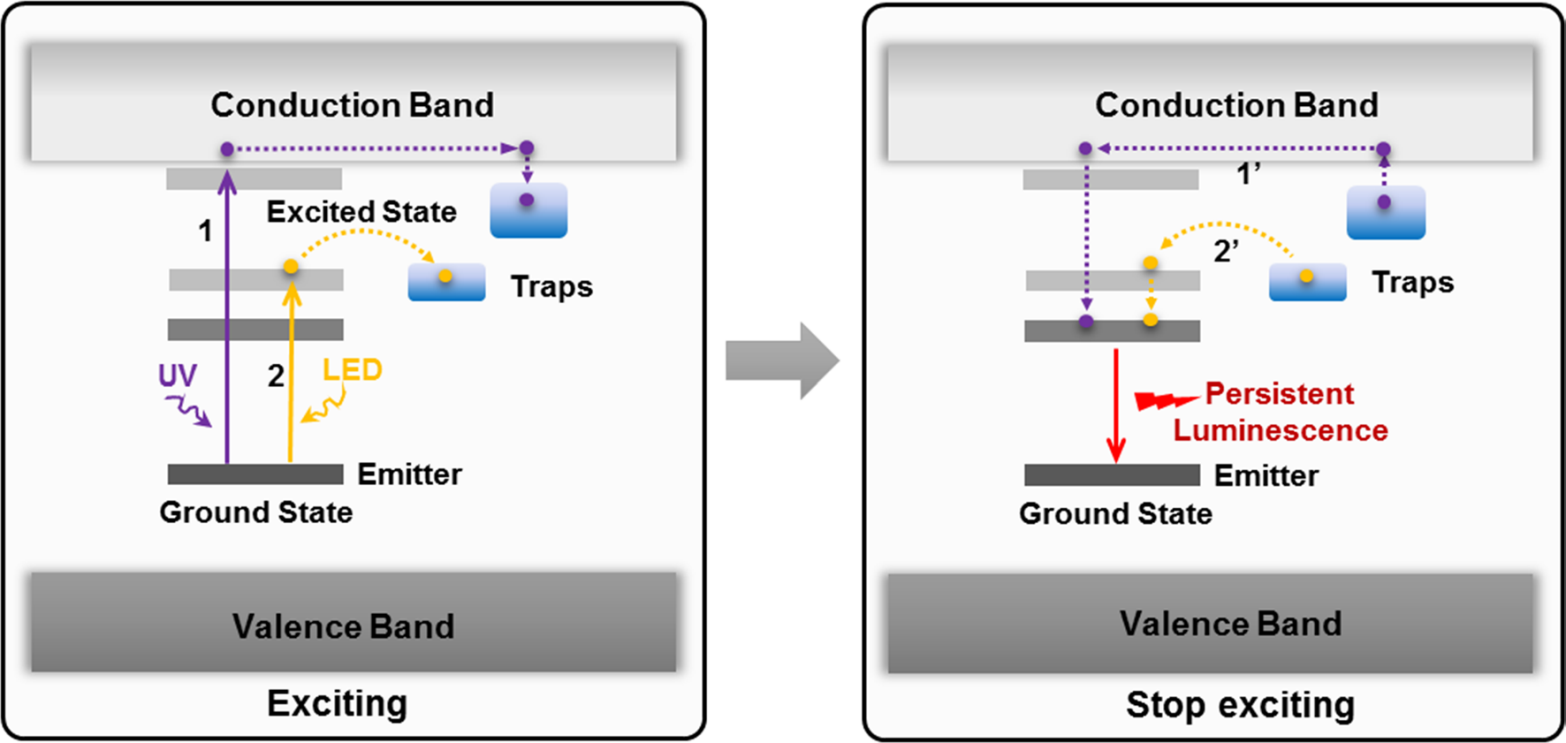
Schematic diagrams of the PersL mechanism under different excitation sources

The mechanism of producing PersL is different under different excitation sources. The widely accepted model of the PersL mechanism is shown in Fig. 2. Under the activation of UV light, the electrons of emitter are excited from the ground state to the conduction band or the excited state near the conduction band, subsequently, the electrons are captured by the traps through the conduction band (process 1). Once ceased the excitation, the electrons escape from the traps and re-enter the conduction band under the stimulation of external factors, recombining with emitter to emit PersL (process 1′) [25]. Upon the stimulus of LED light, the electrons of emitter are excited from the ground state to the corresponding excited state. Subsequently, the electrons are captured by surrounding traps through the quantum tunneling (process 2). Once stopped the stimulation, the electrons in the trap are recombined with emitter through quantum tunneling, emitting PersL (process 2′) [26]. The PersL mechanism of X-ray or 980 nm laser excitation is similar to the above-mentioned mechanisms, except that there is a process of energy transfer under X-ray or 980 nm laser excitation. The excitation energy of X-ray or 980 nm laser is transferred to the emitter through the host or Yb^3+^-Er^3+^/Yb^3+^-Tm^3+^ causing the above-mentioned series of electrons transition, capture, release, and recombination of the emitter, and finally produce PersL [22, 27]. So far, the mechanism for radiopharmaceuticals is still unclear. However, most of radiopharmaceuticals can emit gamma radiation and Cerenkov luminescence during the decay of radionuclides[28, 29], where gamma ray is similar to X-ray but come from different parts of the atom [30], and Cerenkov luminescence have the emission in the range of 250–600 nm [29], thus we speculate that the mechanism of radiopharmaceuticals-excited PersL includes the PersL mechanisms of X-ray, UV light, and LED light.

### Synthesis of PLNPs

The traditional PersL materials are mainly synthesized by solid-state reaction at high temperature [9]. Despite the advantages of high crystallization, strong luminescence, and long PersL time, the irregular morphology and large particle size limit their biomedical application. To obtain nano-sized PLNPs, researchers have been exploring and improving the synthesis methods of PLNPs for more than a decade. At present, the synthesis methods of PLNPs commonly used in biomedical applications mainly include sol–gel method, template method, hydrothermal/solvothermal method, and co-synthesis method (Table 1).

**Table 1:** Comparison of synthesis methods of PLNPs

Synthesis methods	Size (nm)	Morphology	Uniformity	Surfaces group	PersL time	Refs.
Sol–gel method	50–100	Bad	Bad	Lack	Long	[25, 31]
Template method	50–500	Good	Good	Lack	Medium	[32–34]
Hydrothermal/solvothermal method	5–20	Good	Good	Abundance	Short	[35–37]
Co-synthesis method	20–60	Medium	Medium	Lack	Long	[38–40]

Sol–gel method is to first hydrolyze the precursor into sol, and then transform the sol into gel for subsequent calcination (800–1100 °C), resulting in PLNPs with high yield and small size. Scherman et al. synthesized a series of silicate PLNPs by sol–gel method, such as Ca_0.2_Zn_0.9_Mg_0.9_Si_2_O_6_:Eu^2+^,Dy^3+^,Mn^2+^ [31], CaMgSi_2_O_6_:Eu^2+^,Mn^2+^,Pr^3+^ [41], Sr_1.6_Mg_0.3_Zn_1.1_Si_2_O_7_:Eu^2+^,Dy^3+^ [42], Ca_1.86_Mg_0.14_ZnSi_2_O_7_:Eu^2+^,Dy^3+^ [43], Sr_2_MgSi_2_O_7_:Eu^2+^, Dy^3+^[44]. In addition, gallate PLNPs, such as LiGa_5_O_8_:Cr^3+^[45, 46], Zn_2.94_Ga_1.96_Ge_2_O_10_:Cr^3+^,Pr^3+^ and Zn_1.1_Ga_1.8_Ge_0.1_O_4_:Cr^3+^ [25, 47] were also synthesized by sol–gel method, respectively. Sol–gel method has become one of the most commonly used synthesis methods of PLNPs. However, the synthetic products still suffer from inhomogeneous morphology, uncontrollable size, and poor dispersion.

Template method is to use mesoporous silica or carbon nanospheres as a template to bind with precursor ions and then obtain monodisperse, regular morphological PLNPs upon low-temperature calcination. The morphology and particle size of PLNPs can be controlled by the template. Zhang and co-workers have done fruitful work in synthesizing PLNPs by template method. They used mesoporous silica, hollow silica, or carbon nanospheres as a template to controlled synthesize PLNPs, respectively, such as SiO_2_@Zn_2_SiO_4_:Mn[48], SiO_2_@SrMgSi_2_O_6_:Eu_0.01_,Dy_0.02_ [33], SiO_2_@CaMgSi_2_O_6_:Eu^2+^,Pr^3+^,Mn^2+^ [49], SiO_2_@CaTiO_3_:Pr[50], Gd_2_O_3_@mSiO_2_@CaTiO_3_:Pr [51], Zn_1.1_Ga_1.8_Ge_0.1_O_4_:Cr^3+^,Eu^3+^ @SiO_2_[32], mSiO_2_@Gd_3_Ga_5_O_12_:Cr^3+^,Nd^3+^ [34], ZnGa_2_O_4_:Cr^3+^@HMS[52], ZGOCS@MSNs@Gd_2_O_3_ [53], and ZnGa_2_O_4_:Cr^3+^ [54]. These PLNPs have regular spherical morphology, good monodispersity, and 50–500 nm particle size, and NIR PersL, which is very suitable for biomedical applications. Although this method has some limitation to prepare ultrasmall PLNPs, it is still considered as a facile way to make nanocarriers for PersL imaging-guided drug delivery.

Hydrothermal/solvothermal method refers to the preparation of nanomaterials by treating the precursors in a sealed heated solution above ambient temperature and pressure [55]. This method has the advantages of mild synthesis conditions and low agglomeration. Importantly, the synthesized PLNPs have ultrasmall size and facile surface modification. Han et al. report a direct hydrothermal synthesis route for ZnGa_2_O_4_:Cr^3+^, which has 8 nm size and stable colloidal property [56]. Zhang et al. employed this method to synthesize ZnGa_2_O_4_:Cr, Eu [36] and Zn_2_SnO_4_:Cr, Eu [57] which have rich surface groups and ultrasamll-size (< 10 nm). Yuan et al.[37] reported hydrothermal synthesis of Zn_1+x_Ga_2−2x_Ge_x_O_4_:Cr, where the size and PersL are fine-tuned by simply changing the amount of Ge. Although these PLNPs have better advantages in particle size, dispersibility, and surface modification, the prepared PLNPs still face the challenge of weak brightness and short PersL time, which need to be further improved.

Co-synthesis method was reported to synthesize PLNPs with small-size and bright PersL by combining the strengths and weaknesses of each of these approaches. Richard et al. reported the synthesis of ZnGa_2_O_4_:Cr^3+^ by co-synthesis method, which was firstly synthesized the PLNPs precursor by hydrothermal method, and then calcinated at 750 °C to enhance the PersL properties [38]. Yan et al. synthesized the precursor by adding cetyltrimethylammonium bromide (CTAB) into the hydrothermal system as the morphology and particle size control agent [39]. With subsequent calcination of the precursor in a short time, Zn_1.25_Ga_1.5_Ge_0.25_O_4_: Cr^3+^,Yb^3+^,Er^3+^ with around 50 nm of particle size was synthesized, which had good monodispersity and super-long PersL time. Zhang et al. reported a simple EDTA-etching strategy for regulating the size, dispersibility, and PersL of ZnGa_2_O_4_:Cr [58]. The EDTA etching can not only effectively reduce the particle size of PLNPs, but also enhance the aqueous-dispersibility and PersL property. At present, it has been developed as an important synthesis method for PLNPs.

### Surface functionalization of PLNPs

The surface properties of nanomaterials have an important influence on their biomedical applications [59–62]. Most PLNPs are synthesized under calcination, contributing to a lack of modifiable groups on their surfaces. Therefore, surface functionalization of PLNPs is necessary for further biomedical applications. The surface functionalization could endow the following benefits, (i) increase the biocompatibility of PLNPs and reduce their biological toxicity. (ii) increase the stability in physiological solutions and reduce agglomeration. (iii) provide functional groups (e.g. amine, carboxyl) with further biofunctional molecules attachment for enhancing tumor targeting [60]. In this section, we summarize the two commonly used surface functionalization methods for PLNPs: hydroxylation and silicon coating.

Hydroxylation is a commonly used surface modification method for PLNPs, which is mainly achieved by erosion of NaOH on the surface of PLNPs. Richard et al. firstly reported the modification process of surface hydroxylation with NaOH on Ca_0.2_Zn_0.9_Mg_0.9_Si_2_O_6_: Eu^2+^,Dy^3+^,Mn^2+^ and ZnGa_2_O_4_:Cr^3+^, and then do the amino by reacting the hydroxyl groups with (3-Aminopropyl)triethoxysilane (APTES) [38, 63]. This method makes their surface amination for further easily conjugating various biofunctional molecules, such as folic acid (FA) [32], peptide [25], polyethylene glycol (PEG) [64, 65], DNA [37], bovine serum albumin (BSA) [54, 66], hyaluronic acid (HA) [67], and antibody [68]. Hydroxylation has become a standard modification method for various PLNPs.

Silica coating is another important method of surface functionalization of PLNPs. Shen et al. used tetraethyl orthosilicate (TEOS) hydrolysis and CTAB as templates to coat mesoporous silicon on the surface of LiGa_5_O_8_:Cr^3+^ [69]. Wang et al. used the Stöber sol–gel process to coat silica on the surface of ZnGa_2_O_4_:Cr^3+^,Sn^4+^ [40]. Wang et al. used Stöber sol–gel process and hydrothermal method to coat silica on the surface of Zn_1.25_Ga_1.5_Ge_0.25_O_4_:Cr^3+^, Yb^3+^, Er^3+^ [70]. Silica coating provides the good biocompatibility of PLNPs, as well as grants with an easily modified surface, which facilitates the subsequent modification of various biofunctional molecules. To prevent the drug leakage during blood circulation and enhance the tumor-targeting ability, cell membrane vesicles from red blood cells [70], cancerous cells [71, 72], and lactobacillus reuteri [73] are used to camouflage on the silica coating PLNPs, which have the superior abilities of immune escape and tumor adhesion.

### Toxicity studies of PLNPs

The influencing factors of the biological toxicity of nanomaterials mainly include: (i) the stability, (ii) the morphology and particle size, (iii) the surface properties [74–77]. At present, the PLNPs for the biomedical application have biocompatible concerns due to the prolonged retention in normal tissue. Thus, a well understanding of the pharmacokinetics and biosafety issue of PLNPs in biological systems can greatly promote the biomedical applications of PLNPs for future clinical translation.

To date, various cell lines have been used to evaluate the in vitro cytotoxicity of different PLNPs. Most of the results indicated that the PLNPs had no obvious cytotoxicity. Yan and Richard et al. studied the in vitro cytotoxicity of the PEGylated and amination PLNPs, respectively. The results showed that the cancerous cells were exposed to PLNPs at concentrations up to 1 mg/mL for 24 or 48 h without significant effects on cell viability [25, 38]. Zhang et al. exposed the amination PLNPs to different cell lines, and the results of cell viability and apoptosis showed low cytotoxicity of PLNPs [32, 36]. In addition, Zhang et al. selected three types of cells to systematically evaluate the in vitro risk of the PEGylated PLNPs [78]. Results showed that the PEGylated PLNPs had no significant effect on cell viabilities, cell membrane damage, oxidative stress, and apoptosis of three different cell lines.

**Fig. 3:**
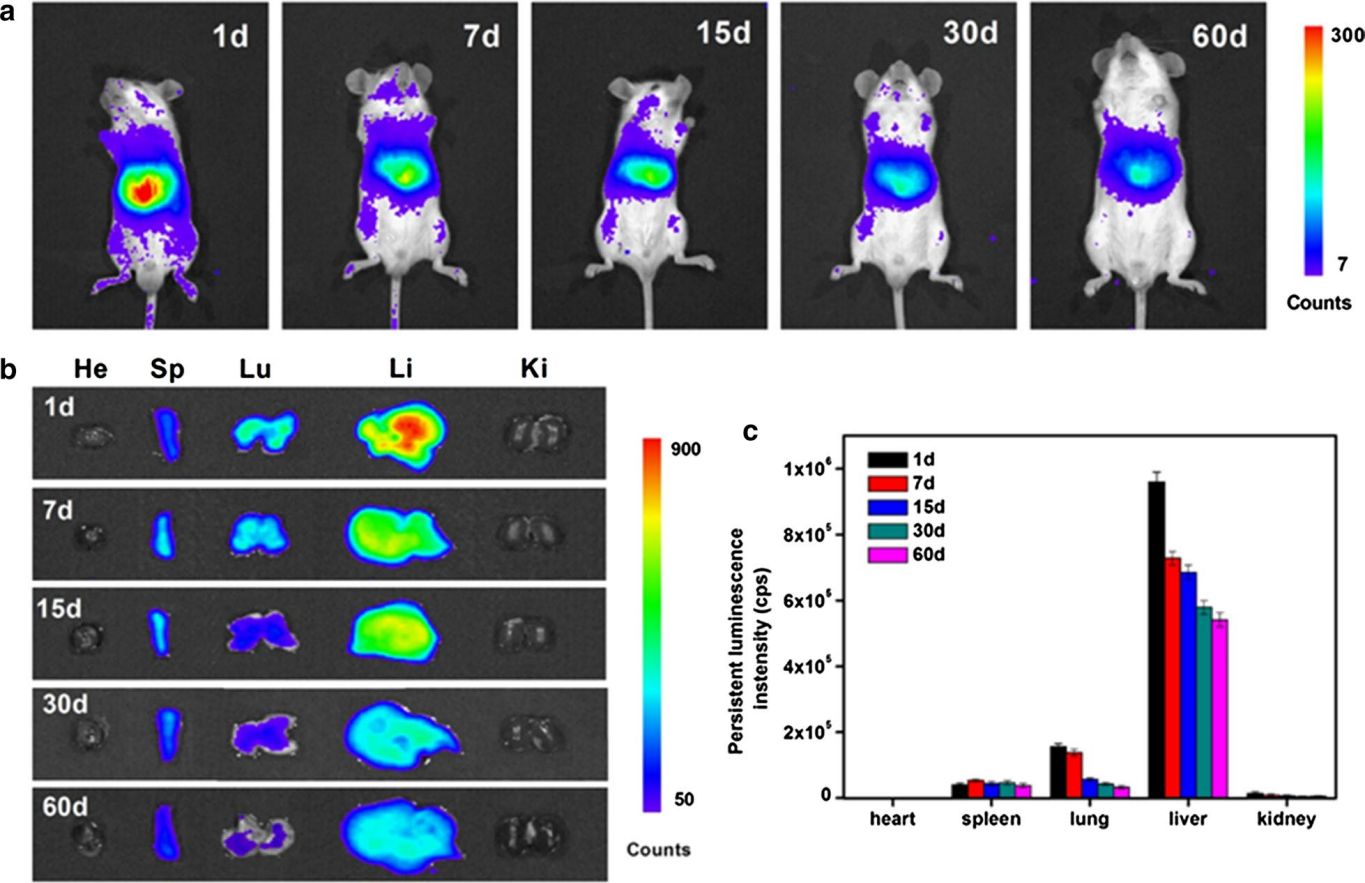
**a** In vivo biodistribution of PEGylated PLNPs at different time points. **b** Ex vivo distribution of PEGylated PLNPs in vital organs at different time points. **c** PersL intensities of PEGylated PLNPs collected from isolated organs at different time points. (Reproduced with permission [78]. Copyright 2018, Nature Publishing Group)

For further applications of PLNPs in the biomedical field, the most important problem is the in vivo biodistribution and toxicity of PLNPs. Liu et al. evaluated the hemocompatibility of pristine PLNPs and PEGylated PLNPs [79]. Results showed that the pristine PLNPs can cause hemolysis, erythrocyte aggregation and morphology changes, and a prolonged coagulation effect, and that these side effects are alleviated by PEGylation. Besides, both pristine PLNPs and PEGylated PLNPs are well tolerated to the risk of complement-activated thrombosis and inflammation. Martínez-Alfaro et al. studied the in vivo toxicity of hydroxylated and PEGylated PLNPs at different concentrations within 6 months [80]. Results showed that no toxic effects were detected at doses of hydroxylated PLNPs ~ 2 mg/mouse classically used for biological imaging. Similarly, no toxic effects could be evidenced on any of the groups treated with PEGylated PLNPs across the range of tested concentrations. The distribution and metabolism of PLNPs in vivo have an important influence on their toxicity. Richard et al. reported the influence of particle size, surface state, and physicochemical properties on PLNPs biological fate in vivo [63, 81]. The results demonstrate that masking charges, increasing the aminosilane density, and reducing the particle size can reduce the capture of PLNPs by the liver and effectively increase the circulation time of PLNPs in vivo. Unlike other fluorescent nanoprobes, the PersL of PLNPs can persist for a long time after excitation, and there is a risk of double exposure of nanoparticles and PersL for in vivo applications. Zhang et al. systematically studied the potential risk of nanoparticles and PersL of PLNPs within 2 months (Fig. 3) [78]. After intravenous injection of 10 mg/kg PEGylated PLNPs, most of them accumulated in the reticuloendothelial system and could be gradually cleared out of the body through the digestive system. Besides, neither the PEGylated PLNPs nor the PersL showed significant toxicity in mice over 2 months. Although more careful toxicology studies are necessary for example the effect of PLNPs on the gene, protein or to evaluate their biotransformation, the lack of obvious toxicity shown in the above studies encourages future development of PLNPs for in vivo biomedical research.

## In vivo bioimaging

### In vivo PersL imaging

Encouraged by the strength of PLNPs, such as the long afterglow, background-free autofluorescence, high sensitivity, and deep tissue penetration, PLNPs are highly suitable for in vivo autofluorescence-free optical imaging. We discuss the in vivo PersL imaging of tumors by different excitation sources, including UV, LED, NIR laser, X-ray, and radiopharmaceutical.

#### UV pre-excitation

**Fig. 4:**
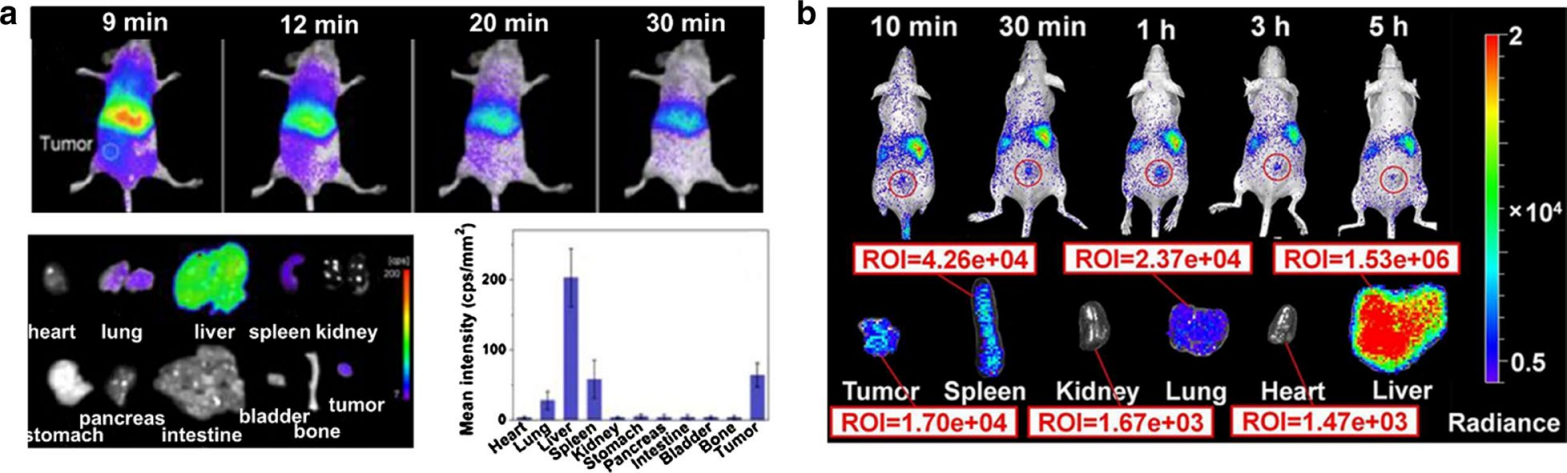
**a** In vivo and ex vivo PersL images of tumor-bearing mice postinjection with RGD-LPLNPs. Reproduced with permission [25]. Copyright 2013 American Chemical Society. **b** In vivo and ex vivo PersL imaging of tumor-bearing mice postinjection with ZGGO:Cr-Apt. (Reproduced with permission [37]. Copyright 2017 American Chemical Society)

In 2007, Scherman et al., for the first time, applied PEGylated Ca_0.2_Zn_0.9_Mg_0.9_Si_2_O_6_:Eu^2+^, Dy^3+^, Mn^2+^ for in vivo PersL imaging of tumor-bearing mice [30]. After pre-excitation of PLNPs by UV lamp, the intravenously injected PLNPs were accumulated to the tumor region in 2 min, which was easily visualized by the PersL signal. Subsequently, Yan et al. synthesized Cr^3+^, Pr^3+^ codoped Zn_2.94_Ga_1.96_Ge_2_O_10_ with almost 15 days of NIR PersL [25]. After surface modification of PEG and tumor targeting molecule RGD, the PLNPs can actively target to tumor region, visualized by high sensitive PersL imaging in 20 min (Fig. 4a). Besides, Yuan et al. constructed DNA aptamer modified Zn_1.2_Ga_1.6_Ge_0.2_O_4_:Cr nanoparticles (ZGGO:Cr-Apt) with long-lasting luminescence and good tumor-specific binding property [37]. Thus the autofluorescence-free targeted imaging of tumors was captured even until 5 h postinjection of ZGGO:Cr-Apt (Fig. 4b). However, due to the slow tumor accumulation of PLNPs and the relatively short afterglow-time of pre-excited PLNPs, thus the in vivo PersL imaging of tumor using UV pre-excited PLNPs usually happens at the beginning time of postinjection, which are not suitable for long-term tracking of tumors.

#### LED in situ excitation

**Fig. 5:**
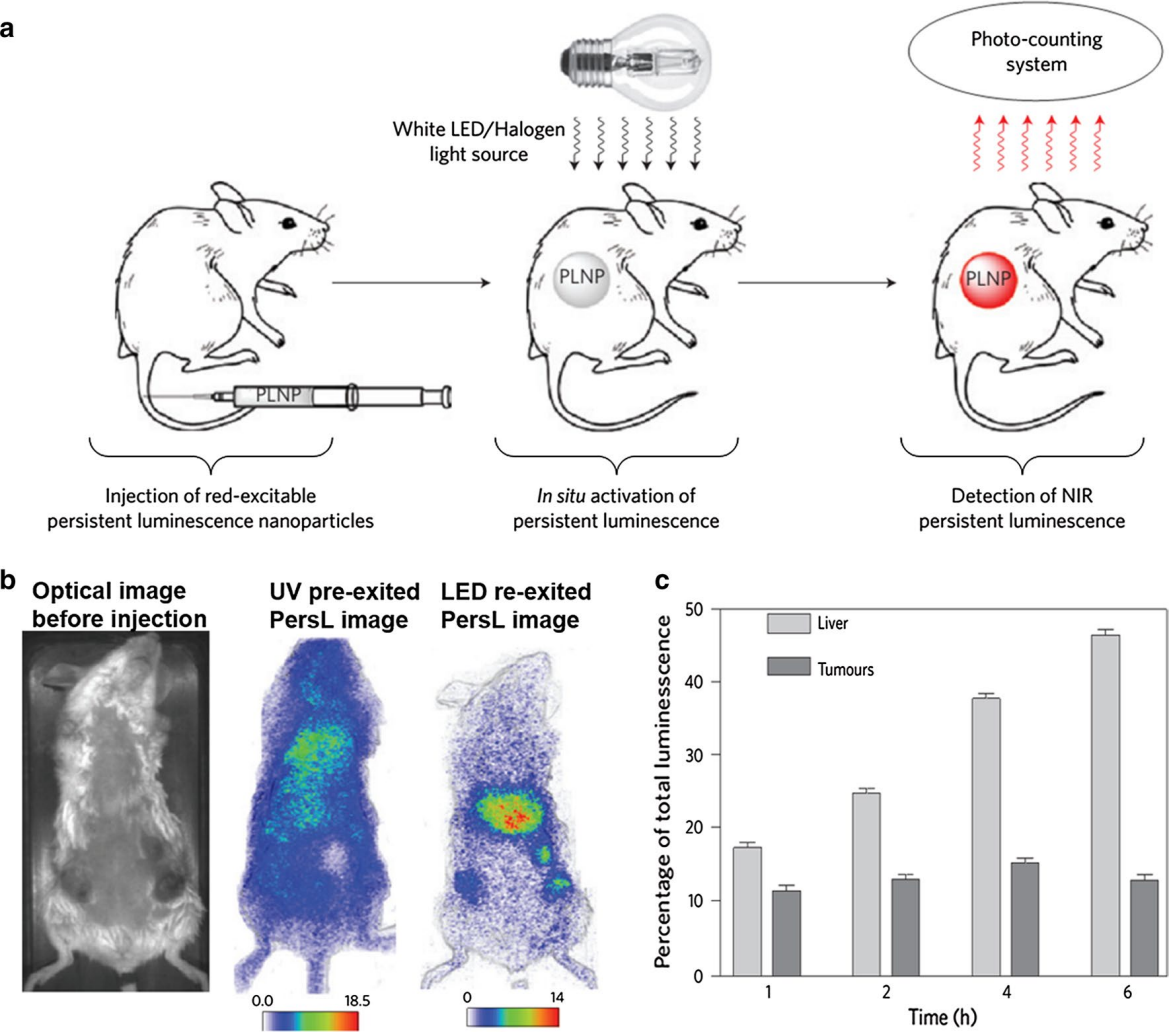
**a** A scheme for in vivo rechargeable PersL imaging based on LED-activated ZnGa_2_O_4_:Cr^3+^. **b** Optical image of a 3-tumor-bearing mouse at preinjection, 2 h post-injection of pre-excited ZGO-PEG, and 4 h post-injection but re-excited with LED. **b** PersL intensities of liver and tumors measured from the whole mice at different time points. (Reproduced with permission [64]. Copyright 2014 Nature Publishing Group)

Richard et al. first employed an orange/red LED as in situ excitation source for in vivo PersL imaging of tumors using PEG-modified ZnGa_2_O_4_:Cr^3+^ nanoparticles [64]. The UV pre-excited PLNPs were intravenously injected for in vivo tumor PersL imaging at 2 h. After the PersL signal of the tumor site is decayed, the red LED was used for *in-situ* re-excitation, which can restore the PersL signal of the tumor site and realize in vivo re-excitation imaging of the tumor (Fig. 5). Later, Pan et al. intravenously injected c(RGDyK) peptide conjugated LiGa_5_O_8_:Cr^3+^ in 4T1 tumor model and used white LED to in situ stimulate tumor accumulated LiGa_5_O_8_:Cr^3+^ for PersL imaging up to 24 h [45]. Besides, Yan et al. reported FA modified Zn_1.25_Ga_1.5_Ge_0.25_O_4_:Cr^3+^,Yb^3+^,Er^3+^ for actively targeting of MCF-7 tumor by oral administration [39]. With the excitation of 650 nm LED, the tumor regions achieve long time (160 min) and high sensitive (SNR > 20) PersL imaging. However, deep tissue imaging is still difficult to be achieved due to the limitations of the LED's own visible wavelength.

#### NIR laser excitation

**Fig. 6:**
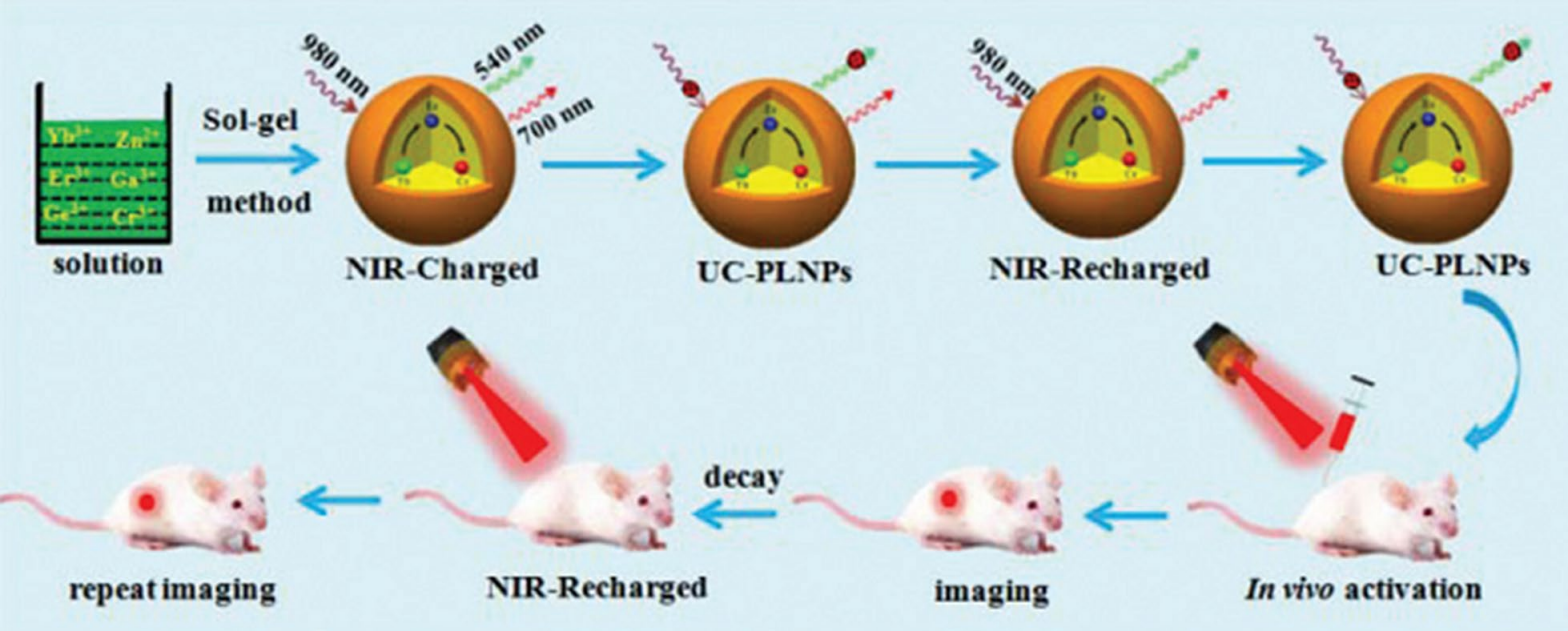
Schematic illustration of the sol–gel method and NIR-to-NIR rechargeable in vivo bioimaging based on 980 nm laser-activated NIR-emitting UC-PLNPs. (Reproduced with permission [85]. Copyright 2017, Royal Society of Chemistry)

The light located in the biological window has better tissue penetration and is suitable for deep tissue reactivation [82, 83]. Therefore, rare-earth doped PLNPs have been developed for NIR laser excitation. Zhang et al. reported that the ultrasmall ZnGa_2_O_4_:Cr,Eu and Zn_2_SnO_4_:Cr,Eu can be easily modified with folic acid molecules through a simple condensation reaction [36, 84]. Highly sensitive targeted imaging of tumor can be achieved by injecting ultraviolet pre-excited PLNPs into mice. After the signal of the tumor site is decayed, the tumor site was re-excited or re-stimulated by the light source of 808 nm with stronger tissue penetration, which can achieve high sensitive tumor imaging. Hao et al. reported a novel 980 nm laser-activated upconverted PLNPs (Zn_3_Ga_2_GeO_8_:Yb/Er/Cr) for in vivo PersL imaging **(**Fig. 6). Owing to the efficient energy transfer (Er^3+^-Cr^3+^), the produced NIR PersL remained up to 15 h. Meanwhile, these upconverted PLNPs also can be effectively recharged in vivo under 980 nm laser’s excitation [85]. Chang et al. developed (Zn_2_SiO_4_:Mn): Y^3+^, Yb^3+^, Tm^3+^ upconverting PLNPs for deep tumor imaging under 980 nm laser [86]. Besides, Li et al. constructed hybrid nanoparticles composed of upconversion nanoparticles and PLNPs, which can be excited by a 980 nm laser and showed PersL emission at 700 nm to realize NIR to NIR upconverted PersL imaging [87]. However, NIR laser as the excitation source for upconverted PLNPs still has some challenges to obtain deep tissue imaging due to the much weaker PersL generated from upconverted PLNPs.

#### X-ray excitation

**Fig. 7:**
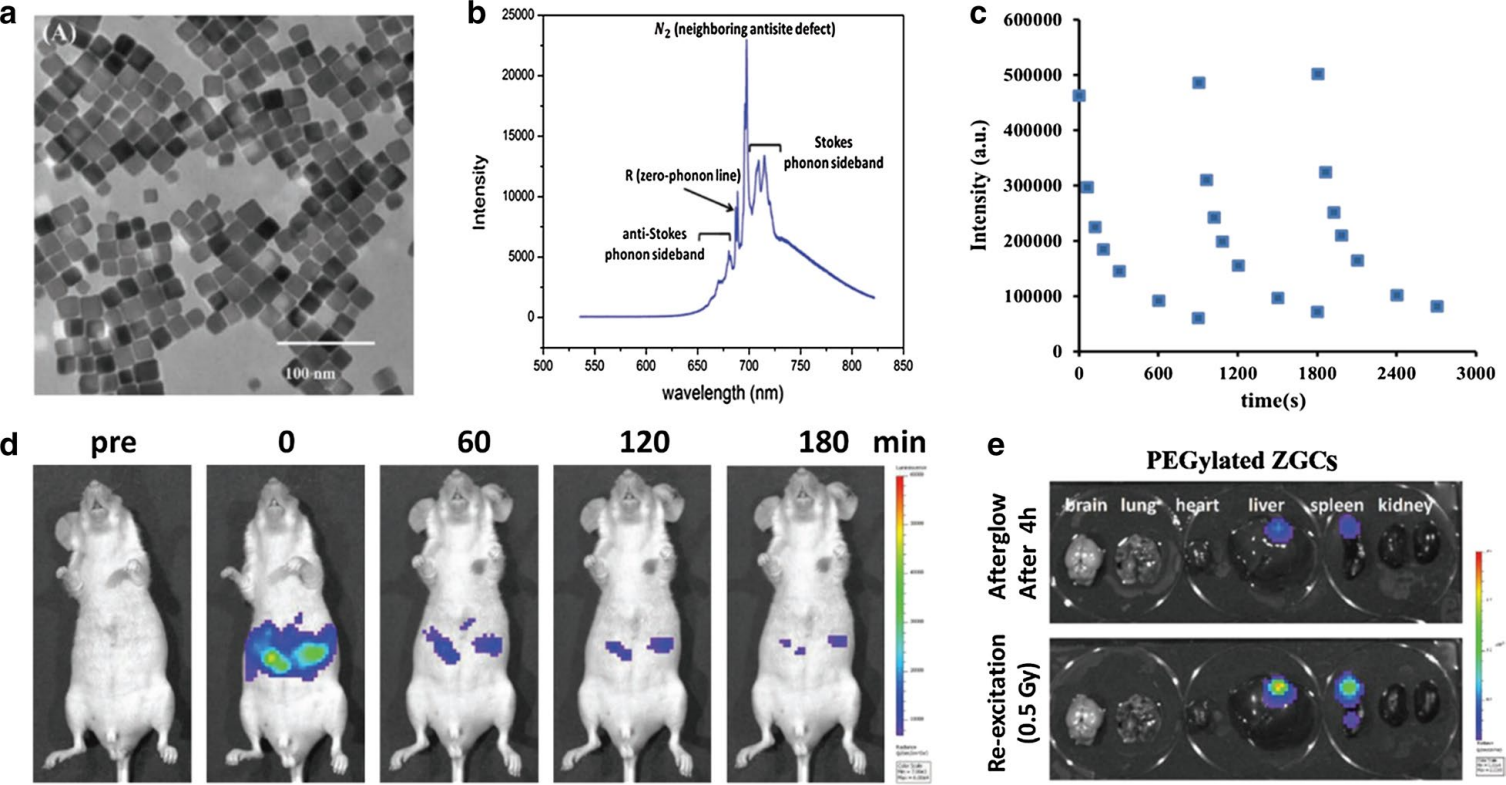
**a**, **b** TEM image and X-ray excited radioluminescence of ZnGa_2_O_4_:Cr^3+^ nanocubes. **c** Rechargeable PersL performance of PEGylated ZnGa_2_O_4_:Cr^3+^ nanocubes under 0.5 Gy X-ray irradiation. **d**, **e** In vivo and ex vivo PersL imaging of tumor-bearing mice treated with iv injected ZnGa_2_O_4_:Cr^3+^ nanocubes and X-ray irradiation. (Reproduced with permission [92]. Copyright 2019, Wiley–VCH)

X-ray has been as an innovative excitation source for in vivo optical imaging in recent years as the excellent merits of negligible scattering and deep depth penetration [88]. Some phosphors can be activated by X-ray photons to generate light by triggering the luminescent centers. Yang et al. firstly proposed X-ray as external optical excitation to activate PEG-functionalized SrAl_2_O_4_:Eu^2+^ PLNPs for imaging deep tissue (up to 2.5 cm) [65]. Hao et al. employed X-ray to activate ZnGa_2_O_4_:Cr PLNPs for renewable NIR PersL imaging of deep-tissue [89]. To synthesize the controllable morphology of PLNPs, Yang et al. developed kiwifruit-like structures of SiO_2_@ZnGa_2_O_4_:Cr@SiO_2_ with the assistant of silica template. Then the X-ray irradiated PLNPs showcased the excellent PersL performance and long-term imaging from deep tissue [90]. Zhang et al. designed MgGeO_3_:Mn^2+^,Yb^3+^,Li^+^ (MGO) PLNPs with NIR-I and NIR-II emission. Under the activation of soft X-rays, MGO can be visualized from deep tissue [91]. Yeh et al. developed PEGylated ZnGa_2_O_4_:Cr^3+^ concave nanocubes with highly passive targeting and X-ray excitation for PersL imaging of deep-seated orthotopic hepatic tumors [92]. The uniform nanocubes showed stable NIR radioluminescence after repeated X-ray excitation (Fig. 7a–c) With the low-dose of X-ray excitation (0.5 Gy), these PEGylated PLNPs clearly depicted the orthotropic hepatic tumors from in vivo and ex vivo PersL imaging (Fig. 7d,e). Despite the superior advantages of deep penetration, the radiation dose from X-ray must be set carefully to avoid radioactive damage in normal tissues.

#### Radiopharmaceutical excitation

**Fig. 8:**
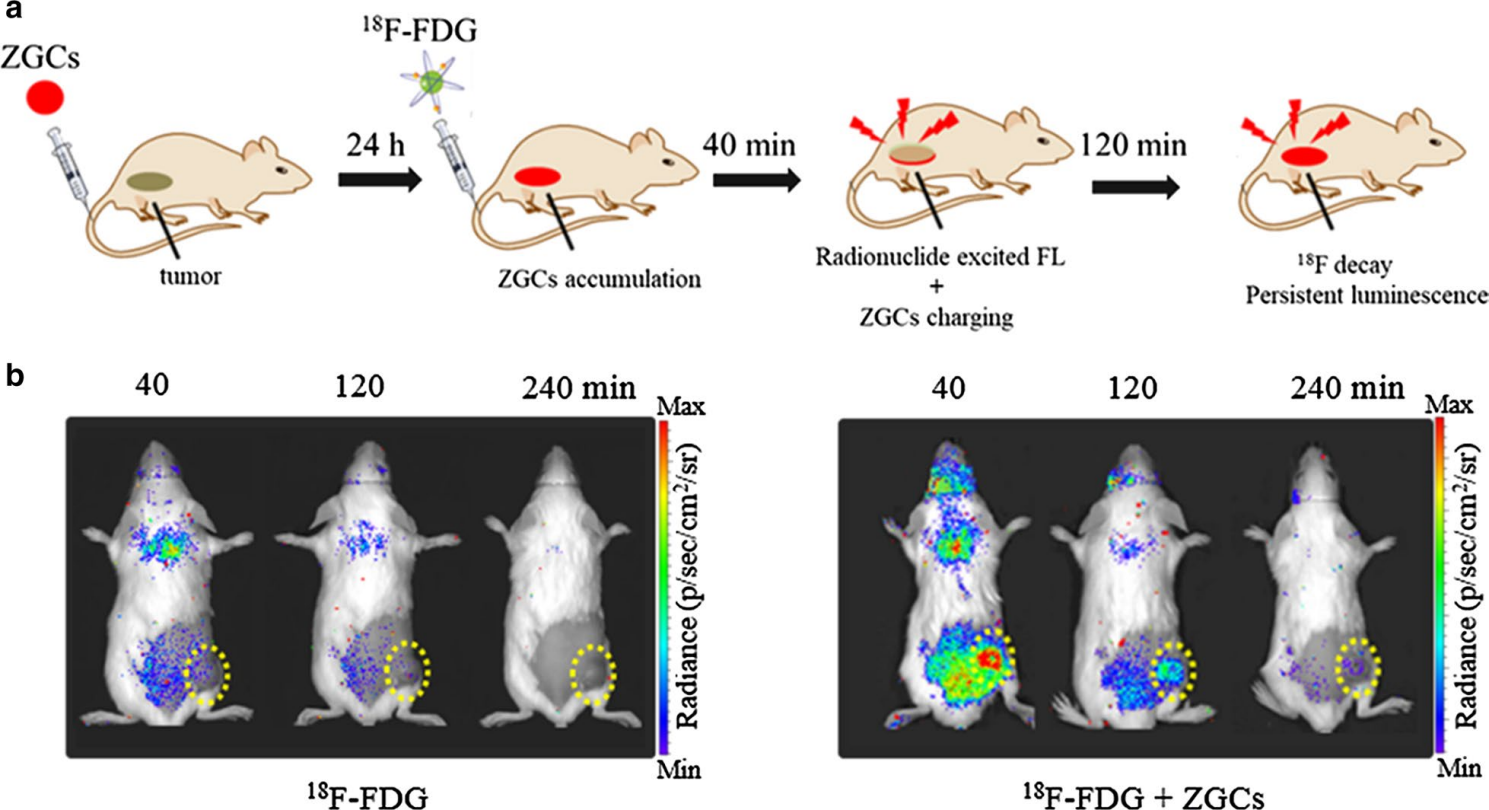
**a** A scheme for in vivo ^18^F-FDG excited PersL imaging of ZnGa_2_O_4_:Cr^3+^. **b** Representative PersL images of 4T1 tumor-bearing mice at different time points after administration of only 200 μCi ^18^F-FDG or 200 μg ZnGa_2_O_4_:Cr^3+^ injection prior 24 h and following with 200 μCi ^18^F-FDG. (Reproduced with permission [28]. Copyright 2020, Wiley–VCH)

Radiopharmaceutical can be regarded as an internal excitation light for deep tissue imaging. Many radionuclides possess Cerenkov radiation with blue light as well as gamma radiation during the process of decay [93]. ZnGa_2_O_4_:Cr^3+^ have a strong excitation spectrum at the UV region and NIR emission, thus we first reported that ZnGa_2_O_4_:Cr^3+^ could be activated by radionuclides with NIR emission for deep tumor imaging, where the NIR emission includes radionuclides’ excited fluorescence and PersL (Fig. 8a) [28]. Then we utilized FDA-approved ^18^F-fluorodeoxyglucose (^18^F-FDG) to in vivo stimulate ZnGa_2_O_4_:Cr^3+^ for optical imaging of tumor. The PersL signal from ^18^F-FDG excited ZnGa_2_O_4_:Cr^3+^ can remain over 3 h at the tumor region while very few luminescence from ^18^F-FDG’s treatment was detected (Fig. 8b). Importantly, ZnGa_2_O_4_:Cr^3+^ could be efficiently recharged in vivo by multiply injection of ^18^F-FDG which enables long-lasting tumor imaging with high sensitivity and high ratio of tumor to liver.

### In vivo multimodal imaging

Multimodal imaging that combines the advantages of different imaging modalities can provide more accurate disease information for precise diagnosis [94]. Therefore, integrating the merits of PLNPs and other image modalities enables to design high-performance PLNPs nanoprobes, such as, X-ray computed tomography (CT), SPECT imaging, magnetic resonance (MR), and photoacoustic imaging (PA) can simultaneously endow physiological information with high spatial resolution, which makes up the limitation from single-modal imaging (PersL).

**Fig. 9:**
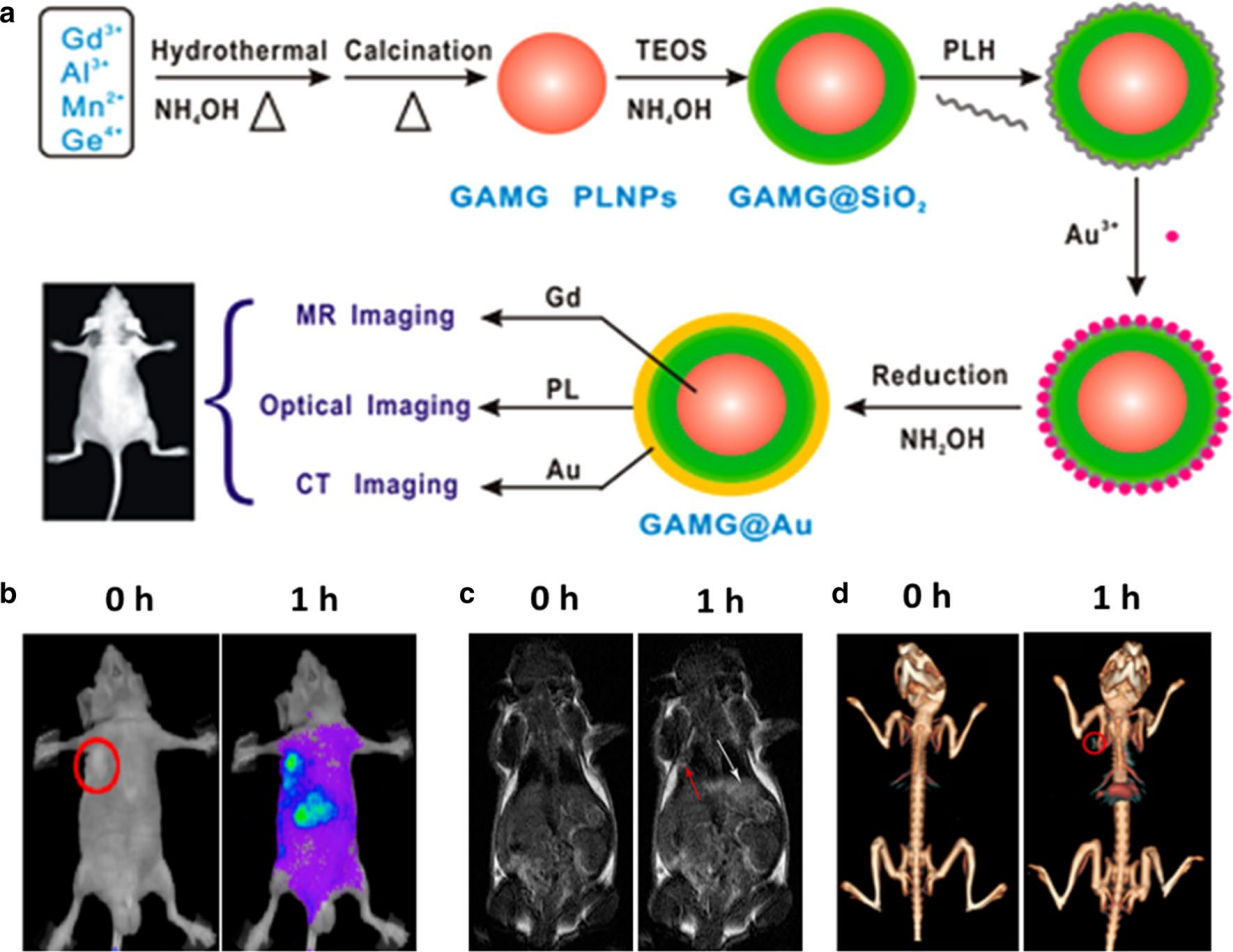
**a** Schematic illustration of the synthesis of GAMG@Au core − shell nanostructure for in vivo tri-modal imaging. **b**–**d** In vivo PersL/MR/CT images before and after intravenous injection of FA-PEG-GAMG@Au. (Reproduced with permission [99]. Copyright 2016 American Chemical Society)

Three major methods have been proposed to construct PLNPs-based multifunctional nanoprobes. The first strategy focuses on the use of surface chemical modifications to attach another imaging modality. For example, Gao et al. developed c(RGDyK) peptide and radioisotope ^99m^Tc labeled PLNPs for targeted PersL/SPECT imaging of orthotopic breast cancer after oral administration [95]. Yan et al. reported Gd-DTPA modified PLNPs (Gd(III)-PLNPs) for in vivo NIR persistent luminescence and T1-weighted MRI imaging [96]. Besides, Yan et al. employed hyaluronic acid-functionalized Gd_2_O_3_ (HA-Ga_2_O_3_) to conjugate on PLNPs [67]. The conjugation not only had the tumor active-targeting capability but also exhibited strong MR and PersL signals in tumor regions. The second method is to introduce the core–shell structured PLNPs by sequential growth or coating. Yan et al. reported the multifunctional core–shell nanostructures (Zn_2.94_Ga_1.96_Ge_2_O_10_:Cr^3+^,Pr^3+^@TaO_x_@SiO_2_) for in vivo PersL/CT imaging of tumor [97]. Zhang et al. constructed polypyrrole-coated PLNPs which offered dual-modal PersL/PA imaging of tumors [98]. Wang et al. also presented GdAlO_3_:Mn^4+^,Ge^4+^@Au (GAMG@Au) core–shell nanoprobes with MR/CT/PersL third-modals imaging properties [99]. After modified with folic acid-PEG-SH, the nanoparticles could actively accumulate at the tumor regions, which were clearly visualized by MR/CT/PersL imaging (Fig. 9). However, this core–shell strategy brings an inevitable size increase and a decreasing PersL owning to the photon reflection or assimilation from shell structure. Finally, the doping approach is much preferable because of the simple preparation and stable physicochemical property. Richard et al. synthesized ZnGa_1.915_Cr_0.005_Gd_0.08_O_4_ nanoparticles by sintering in the air after hydrothermal crystallization, which allowed the high-sensitive optical detection and high-spatial-resolution MR imaging in vivo [100]. Recently, Song et al. also employed the co-synthesis method to prepare Bi_2_Ga_4_O_9_:Cr PLNPs, which enabled X-ray excited PersL imaging as well as Bi-enhanced CT imaging [101].

## PLNPs based cancer therapy

### PLNPs based surgery

**Fig. 10:**
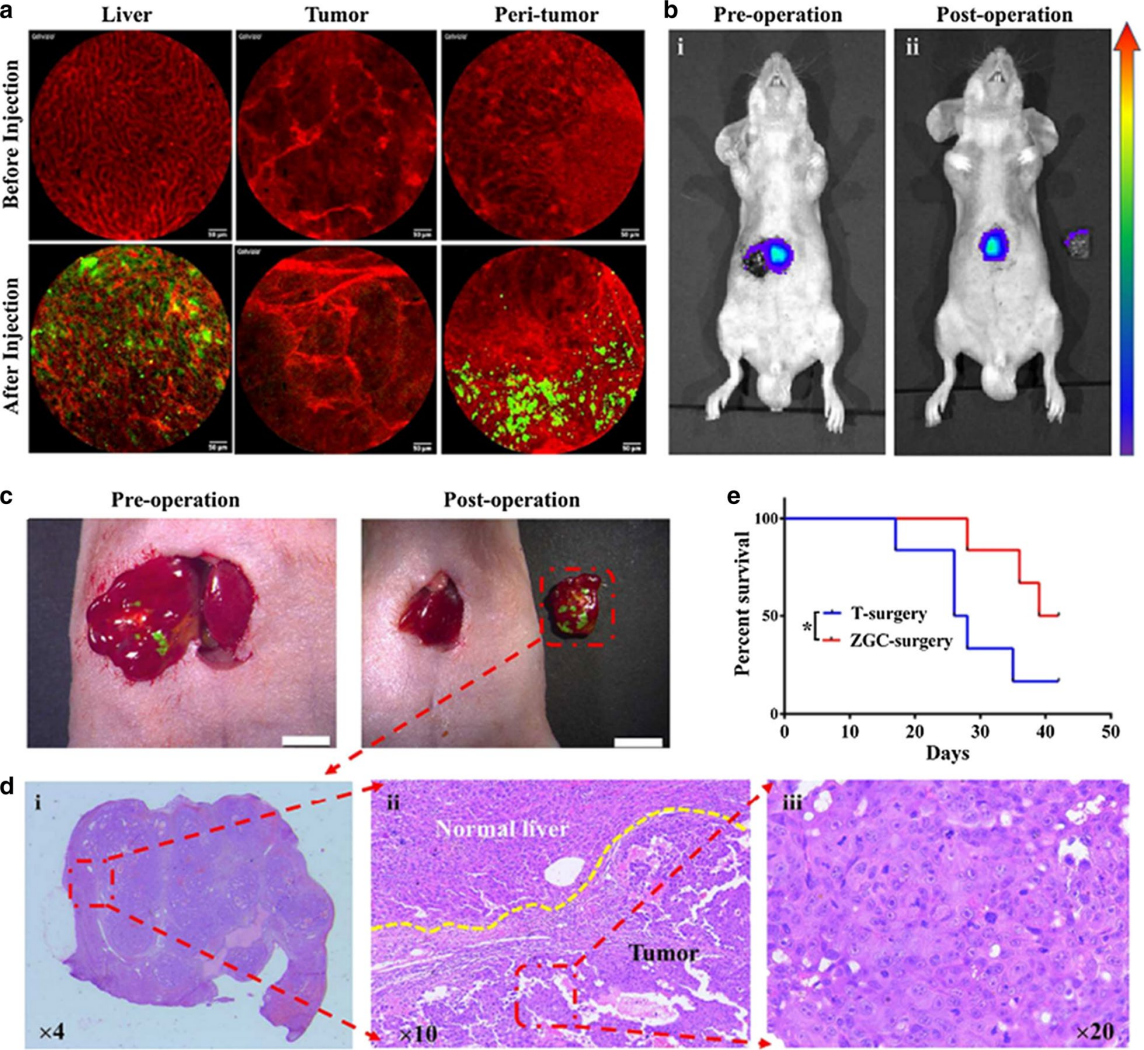
**a** Confocal laser endomicroscopic images of liver, tumor, and peri-tumoral area at 6 h post-injection of ZnGa_2_O_4_:Cr^3+^. **b** PersL images of the exposed tumor lesion following the PLNPs’ injection and LED’s irradiation. **c** GFP fluorescence images of the 3 exposed tumor lesions were merged with the white-light images. **d** H&E staining of the resected tissue from **c**. **e** The survival rates of mice from different treatments. (Reproduced with permission [102]. Copyright 2018, Elsevier)

Fluorescence-guided surgery (FGS) uses the real-time fluorescence images of disease to guide surgical operation, which paves a much cheaper and easier way for precise resection of tumors [37]. FGS can provide real-time imaging during surgery, which is much cheaper and much easier to operate compared to conventional imaging technologies. Due to the excellent advantages of PLNPs (NIR emission, long PersL, and high signal-to-background ratio), Tian et al. employed ZnGa_2_O_4_:Cr^3+^ for the long-term image-guided surgery of hepatocellular carcinoma (HCC) [102]. These ZnGa_2_O_4_:Cr^3+^ were prepared following a reported method [56]. Interestingly, there was no uptake of ZnGa_2_O_4_:Cr^3+^ by HCC tumor tissue but a significant uptake by normal liver tissue, allowing for a precise mapping of the HCC tumor area with more radical excision **(**Fig. 10).

### PLNPs based photothermal therapy

**Fig. 11:**
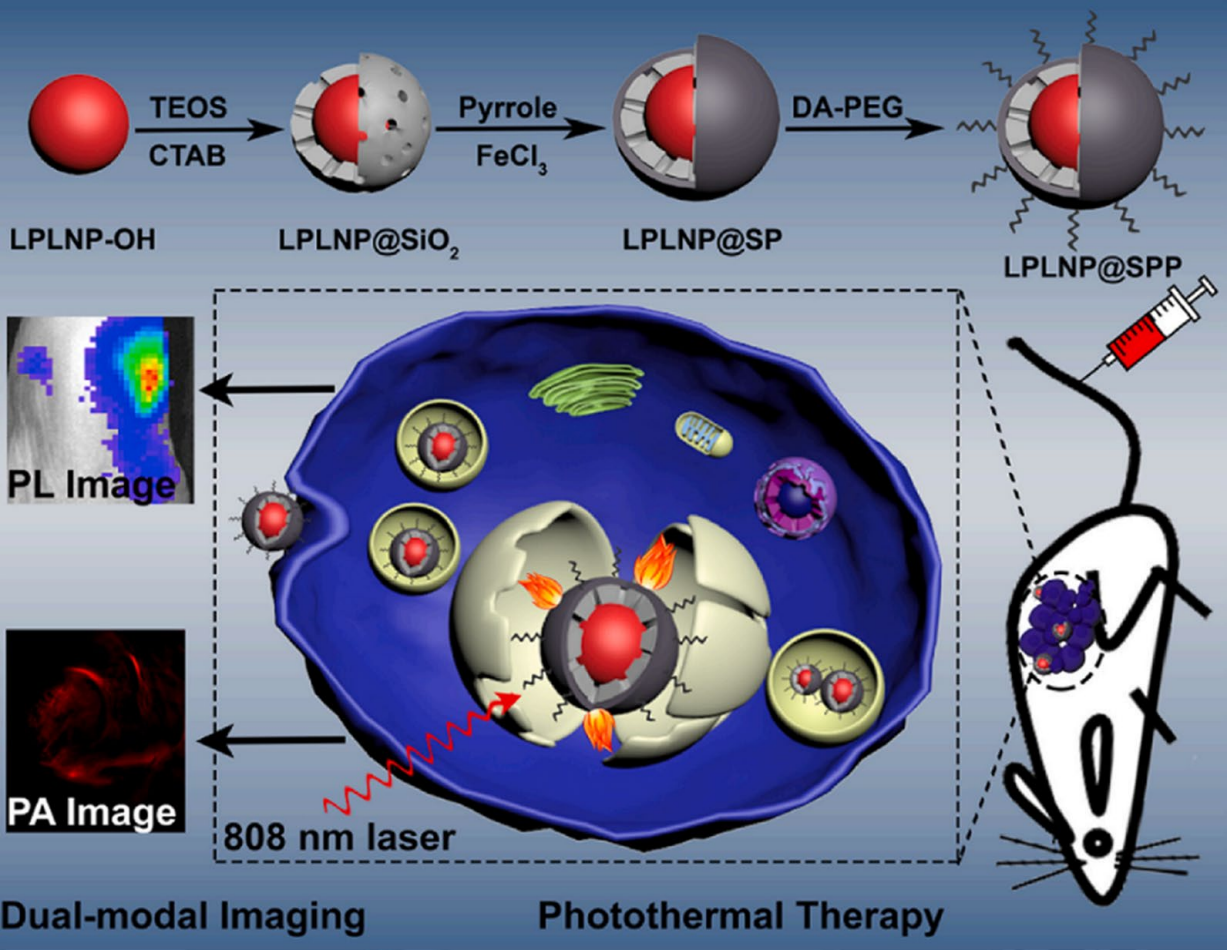
Schematic diagram of LPLNP@SPP synthesis method, dual-modal imaging-guided PTT. (Reproduced with permission [98]. Copyright 2021, Elsevier)

Photothermal therapy (PTT) uses photo-absorbers that absorbs laser energy to produce enough heat to kill cancer cells. Because of the superiority of minimal-invasiveness and spatial specificity, photo-absorbers based PTT has been used in numerous pre-clinical studies [103]. Although PLNPs themselves cannot be used directly in photothermal therapy due to their low extinction coefficient, coupling with NIR absorbing materials (such as ICG, CuS) can be achieved for PersL imaging-guided PTT of tumors. Chang et al. designed the PLNPs and ICG co-loaded mesoporous silica for PersL imaging-guided PTT [104]. The constructed nanoplatform had strong NIR absorption with an excellent photothermal response, which showed efficient tumor elimination in vitro and in vivo. Yan et al. developed an activatable PLNPs/CuS-based nanoplatform for PersL imaging-guided PTT [105]. CuS nanoparticles were regarded as both PTT agents and quencher to afford the high photothermal conversion efficiency to the whole nanoformulation. Thus the synthesized nanoplatform exhibited highly sensitive PersL imaging of tumors and excellent tumor treatment. Zhang et al. also reported polypyrrole-coated PLNPs which enabled good PersL/PA imaging and efficient photothermal effect on tumor inhibition (Fig. 11) [98].

### PLNPs based photodynamic therapy

Photodynamic therapy (PDT) has been widely used in clinical research and practice to treat most solid tumors due to its non-invasiveness and double selectivity [106, 107]. In general, external light is employed to activate PSs, producing reactive oxygen species (ROS) and damaging cancer cells. However, a major challenge of PDT is the requirement of visible or even UV light for PSs’ excitation, where these short-wavelength light sources have limited penetration depth and strong scattering in vivo, leading to the low efficiency of tumor treatment [108]. PLNPs can act as nanocarriers of PSs to achieve effective treatment of tumors, because PLNPs can be excited by other light sources (LED, NIR laser, X-ray, and radiopharmaceuticals) to produce PersL, which in turn activates photosensitizers of corresponding absorption wavelengths and finally produces continuous ^1^O_2_ to kill cancer cells. Besides, PLNPs remain long-lasting PersL for continuous PSs activation after removing away the excitation source, which can avoid the side effects induced by the prolonged irradiation. Furthermore, PLNPs aid in the loading and delivery of PSs because of the easy surface modification for PSs loading and tumor targeting (Table 2).

**Table 2:** A summary of published works about PLNPs-based PDT

PLNPs	Surface coating	Photosensitizers	Attached strategy	Loading capacity	Excitation source	Operated subject	Refs.
Zn_1.25_Ga_1.5_Ge_0.25_O_4_: Cr^3+^, Yb^3+^, Er^3+^	Mesoporous silica	AlPcS	pore loading	3.2 wt%	UV light	Cells	[109]
Hollow ZnGa_2_O_4_:Cr^3+^	BSA	Si-Pc	Pore loading	850 mg/g	LED	Animal (iv)	[54]
Zn_2_Ga_2.98_Ge_0.75_O_8_:Cr^3+^_0.02_,Bi^3+^_*x*_	Mesoporous silica	ZnPc	Pore loading	0.346 wt%	Red light	Animal (it)	[110]
ZnGa_2_O_4_:Cr^3+^	Oleic acid and hexadecanol	IR780 iodine	Wax-sealed	33.7 ± 2.8 wt%	LED	Animal (iv)	[111]
ZnGa_1.996_O_4_:Cr^3+^	PLGA/NMP oleosol	HPPH	–	–	LED	Animal (it)	[112]
Zn_1.25_Ga_1.5_Ge_0.25_O_4_: Cr^3+^, Yb^3+^, Er^3+^	Alginate-Ca^2+^ hydrogel	Chlorin e6	–	–	Red light	Animal (it)	[113]
Zn_3_Ga_2_GeO_8_:Cr^3+^	Silylation	Si-Pc	Covalent binding	–	808 nm laser	Animal (it)	[114]
NaYF_4_:Yb^3+^,Tm^3+^ SrAl_2_O_4_: Eu^2+^,Dy^3+^	Polydimethylsiloxane	Rose Bengal	Hydrogel loading	1.565 mg/g	980 nm laser	Animal (it)	[115]
ZnS:Cu,Co		TBrRh123	Amidation cross-linking	5 wt %	X-ray	Cells	[116]
SrAl_2_O_4_:Eu^2+^	Mesoporous silica	MC540	Pore loading	15 wt %	X-ray	Animal (it)	[117]
LiGa_5_O_8_:Cr^3+^	Mesoporous silica	2,3-Naphthalocyanine	Pore loading	2 wt%	X-ray	Animal (iv)	[69]
ZnGa_2_O_4_:Cr/W	Silylation	ZnPcS4	Covalent binding	32.25 µg/mg	X-ray	Animal (iv)	[119]
Mesoporous Zn_3_Ga_2_GeO_8_:Cr^3+^,Yb^3+^,Er^3+^	PEG modification	Si-Pc	Pore loading	29.7 wt%	X-ray	Animal (iv)	[118]

**Fig. 12:**
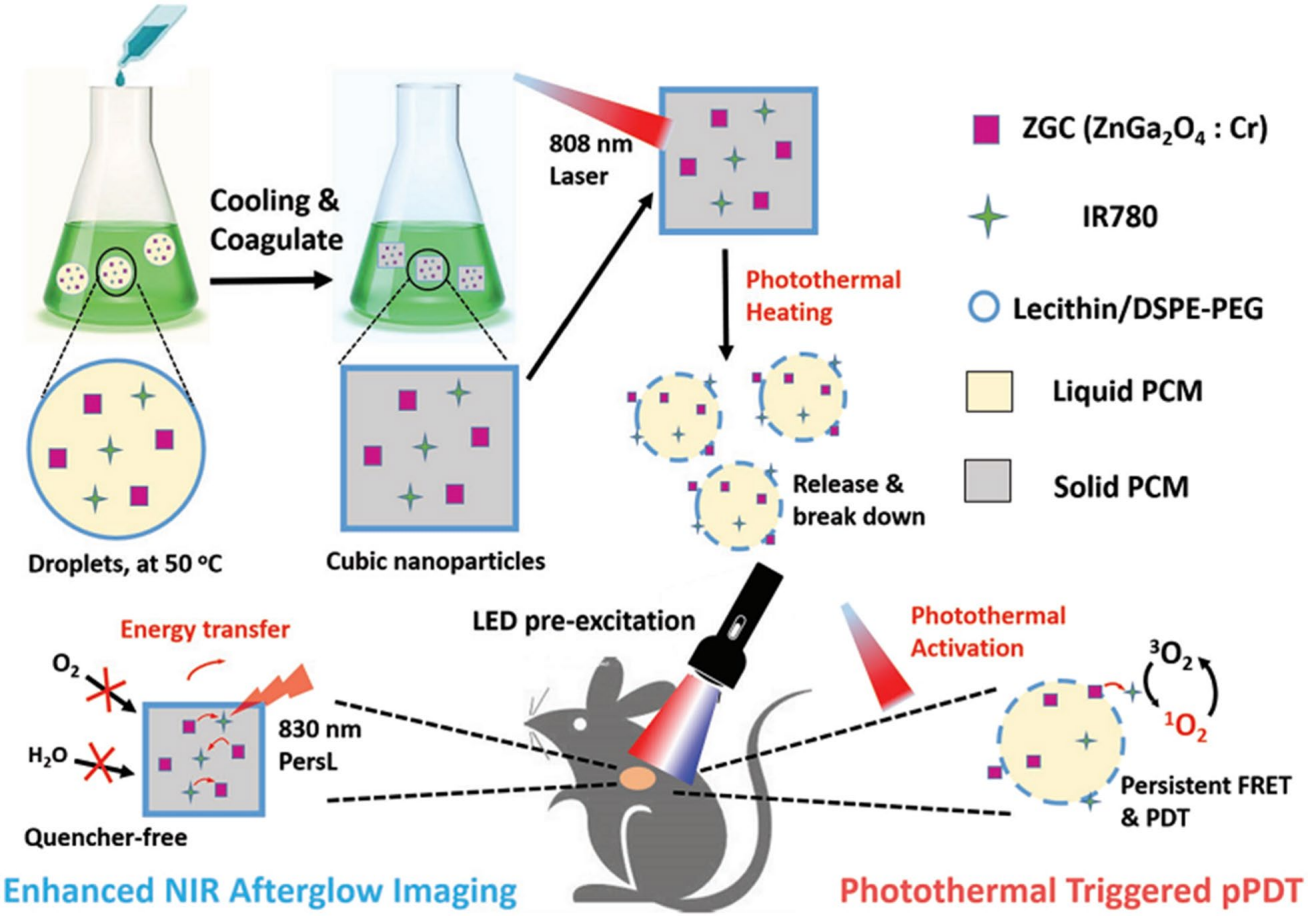
A scheme of the “wax-seal” design of IR-ZnGa_2_O_4_:Cr^3+^ nanoplatform for enhanced PersL imaging and photothermal-triggered persistent PDT. (Reproduced with permission [111]. Copyright 2019, Wiley–VCH)

Liu et al. prepared sulfonated aluminum phthalocyanine (AlPcS) conjugated Zn_1.25_Ga_1.5_Ge_0.25_O_4_:Cr^3+^,Yb^3+^,Er^3+^ @mSiO_2_ for UV excited PDT of cancer cells [109]. Due to the unavailability of UV excitation for in vivo study, some groups tried to use commercial LED light to excited PLNPs for PSs’ activation. Zhang et al. designed hollow ZnGa_2_O_4_:Cr with high silicon phthalocyanine (Si-Pc) loading for LED excited in vivo PDT [54]. Liu et al. developed Bi^3+^ and Cr^3+^ codoped zinc gallogermanate nanoparticles with enhanced deep red PersL emission and PersL time. After coated with mesoporous silica and loaded Zinc phthalocyanine (ZnPc), red light endowed this nanoplatform for direct excitation of PSs and later discharging PersL’s excitation of PSs [110]. Dong et al. proposed to encapsulate ZnGa_2_O_4_:Cr^3+^ and IR780 iodine into a temperature-responsive “waxseal” for imaging-guided and localized PDT [111]. The waxseal could prevent luminescence quenching as well as premature initiation of PDT. After photothermal activation, the NIR PersL from PLNPs not only provided high sensitive images of tumors but also continuously excited PSs for reactive oxygen species generation (Fig. 12). To achieve long-term repeatable PLNPs-excited-PDT, PersL implants are proposed to maintain high-dose of PLNPs within tumors. Chen et al. designed injectable PersL implants as an internal excitation source for repeatable LED plus NIR PersL-excited PDT [112]. The implants were synthesized by dissolving ZnGa_2_O_4_:Cr^3+^ in PLGA/NMP oleosol, which enabled the repeated “charging” process by LED excitation. The LED and PersL-induced-PDT efficiently activated a tumor-sensitive HPPH for ROS generation and remarkably improved therapeutic effects. Yu et al. developed a facile “turning solid into hydrogel” strategy to make full use of PersL for high-efficient PDT [113]. The PersL-hydrogel was simply prepared by mixing PLNPs into a biocompatible alginate-Ca^2+^ hydrogel. Then the PL-hydrogel offered intact and renewable PL for continuous PDT of tumors.

To enhance the excitation depth, the longer-wavelength excitation could be the potential excitation source. Yan et al. reported the covalent coupling of Si-Pc onto Zn_3_Ga_2_GeO_8_:Cr^3+^ for 808 nm laser repeated PersL-sensitized long-term PDT of tumors [114]. The PLNPs were excited by 808 nm laser with 694 nm PersL emission for SiPc’s activation. Zhang et al. reported a NIR rechargeable “optical battery” implant for irradiation-free PDT by loading NaYF_4_:Yb^3+^,Tm^3+^, SrAl_2_O_4_: Eu^2+^,Dy^3+^, rose bengal into biocompatible polydimethylsiloxane (PDMS) [115]. In such a system, 980 nm NIR laser can be firstly excited NaYF_4_:Yb^3+^,Tm^3+^ with UV/blue emission, then the activated SAO, in turn, emits green light to trigger rose Bengal for ROS generation. The implants can be repeatably charged by 5 s NIR light but for 30 min effective PDT time, which effectively generate ROS for tumor inhibition.

**Fig. 13:**
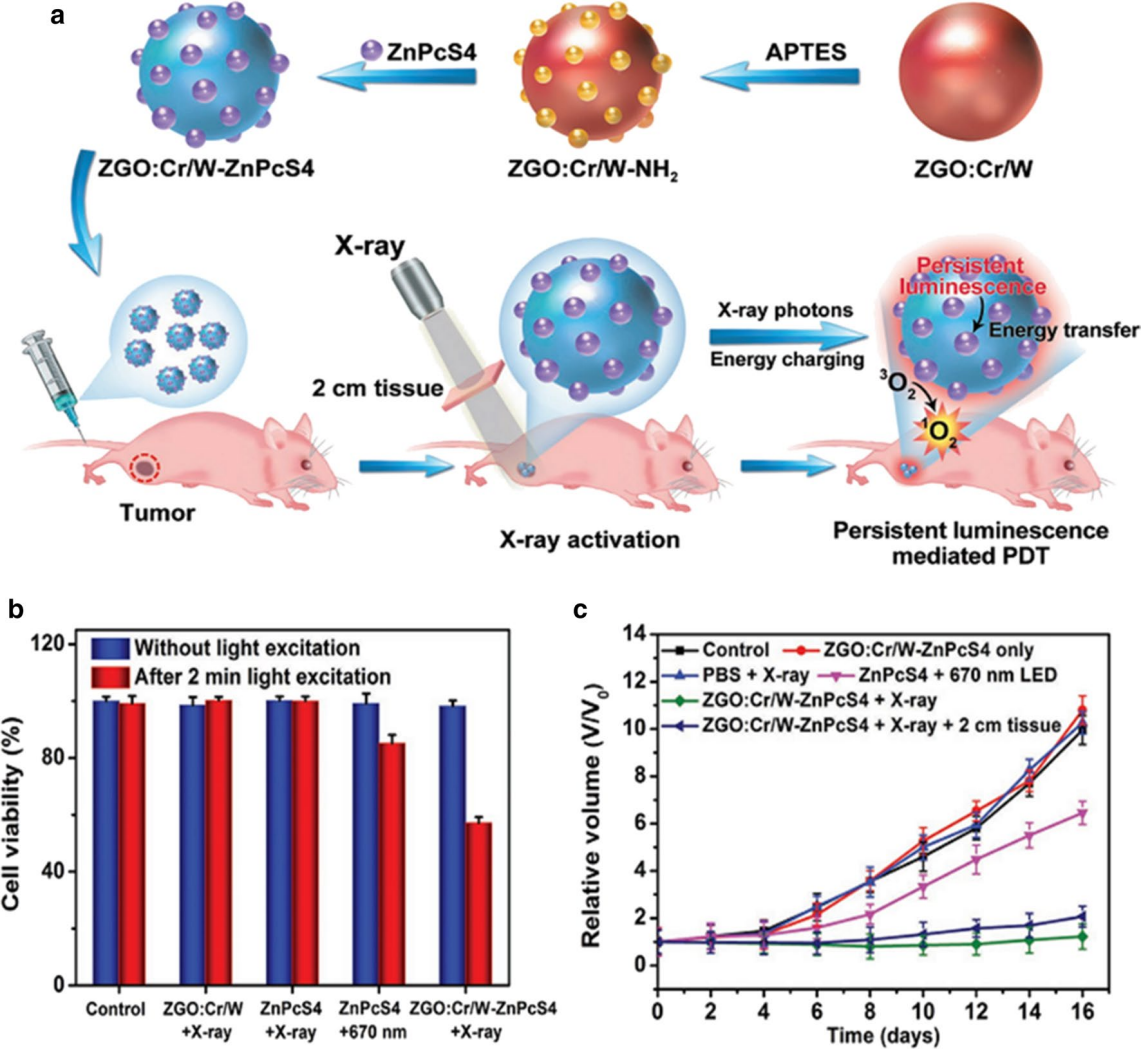
**a** Schematic illustration for the design of X-ray activated PLNP-mediated PDT nanoplatform. **b** Viabilities of HeLa cells with different treatment. **c** Tumor growth curves of different groups of tumor-bearing mice after various treatments. (Reproduced with permission [119]. Copyright 2019, Wiley–VCH)

Inspired by the superior penetration depth of X-ray for the activation of PLNPs, Solberg et al. first employed PLNPs as the photon transducer to achieve X-ray-induced PDT [116]. He used Tetrabromorhodamine-123 (TBrRh123) to conjugate on ZnS:Cu,Co PLNPs, where the emission spectrum of ZnS:Cu,Co was overlapped with the absorption spectrum of TBrRh123. After the X-ray excitation, the nanoplatform continuously generated ROS for human prostate cancer cells killing. Later, Xie et al. reported Merocyanine540 (MC540)-loaded silica-coated-SrAl_2_O_4_:Eu^2+^ (SAO) nanoplatform for in vivo PDT, where the SAO could effectively convert X-rays photons to visible photons for activating MC540 to generate ^1^O_2_ and suppress tumor growth [117]. However, this therapeutic effect was achieved with intratumorally injected nanoparticles on subcutaneous tumor models. Then Xie et al. later reported 2,3-naphthalocyanine and LiGa_5_O_8_:Cr^3+^ co-loaded mesoporous silica nanoparticles (NC-LGO:Cr@mSiO_2_) for PDT of H1299 orthotopic lung cancer [69]. The nanoformulation was passively accumulated to lung tumors. Upon X-ray’s irradiation, the tumors were obtained efficient inhibition. Furthermore, Chen et al. reported another nanoplatform by loading silicon phthalocyanine into mesoporous Zn_3_Ga_2_GeO_8_:Cr^3+^,Yb^3+^,Er^3+^ (mZGGOs) for X-ray-induced PersL imaging and effective suppression of orthotopic hepatic tumors [118]. Due to the safety concern of high-dose X-ray, Yang et al. developed a low-dose X-ray-activated PLNP-mediated PDT nanoplatform for renewable cancer treatment (Fig. 13a) [119]. The synthesized ZGO:Cr/W exhibited stronger PersL and excellent X-ray absorption, allowing for more photons to activate Zn(II) phthalocyanine tetrasulfonic acid (ZnPcS4). Besides, 0.18 Gy X-ray’s irradiation for this nanoplatform also produced enough PDT effect for the treatment of deep-seated tumor (Fig. 13b,c).

### PLNPs based chemotherapy

**Fig. 14:**
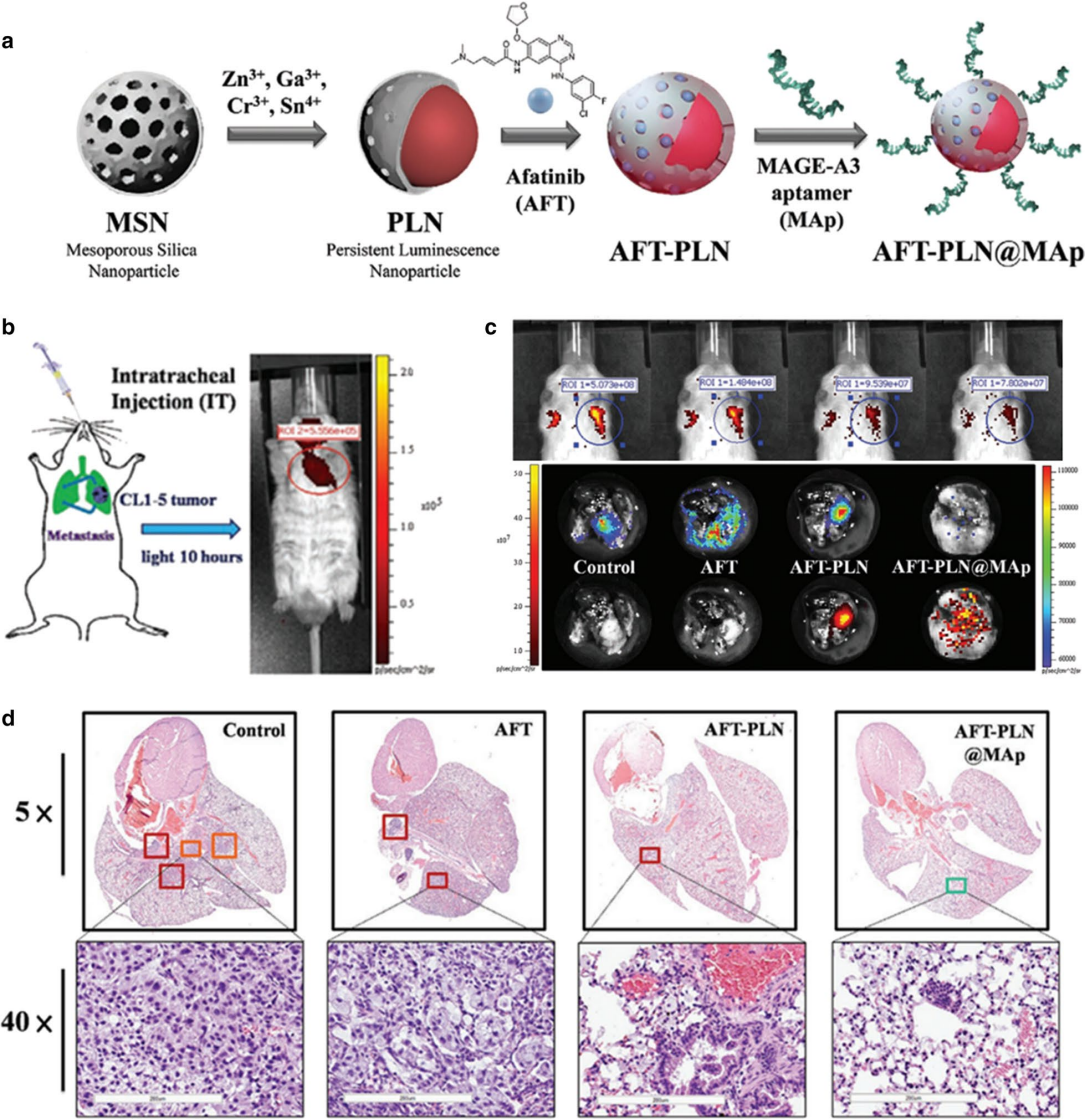
**a**, **b** Schematic synthetic procedures for AFT-PLN@Map and the intratracheal injection of UV pre-excited AFT-PLN@Map. **c** PersL imaging after 6 h of different treatment, and the corresponding PersL and fluorescence imaging of the isolated lung. **d** H&E staining of isolated lungs from various treated groups. (Reproduced with permission [120]. Copyright 2020, Wiley–VCH)

Nanocarriers can enable the loading of multiple chemotherapeutic drugs while enhancing efficient drug delivery to tumors. Currently, PLNPs with different nanostructure have been explored as nanocarriers for chemodrugs loading and delivery. Several design strategies have been proposed on PLNPs, mainly resting on the physical absorption of porous nanostructure (Table 3). Firstly, PLNPs with their own mesoporous structure have attracted considerable attention for drug delivery because of their high cavity volumes and specific surface area. For example, Zhang et al. designed hollow ZnGa_2_O_4_:Cr^3+^ with high doxorubicin (DOX) loading for PersL imaging-guided chemotherapy [54]. Lv et al. developed raspberry-like mesoporous Zn_1.07_Ga_2.34_Si_0.98_O_6.56_:Cr_0.01_ nanostructures for enhanced PersL imaging and chemotherapy of tumor [66]. These two mesoporous PLNPs nanostructures both had excellent high drug loading efficiency, and the authors utilized BSA to modify the structures to improve biocompatibility and colloidal stability. Besides, mesoporous silica shells coating on PLNPs can be another strategy for drug loading. Zhang et al. employed mesoporous silica nanospheres (MSNs) both as morphology-controlling templates and as drug carriers to design porous PLNPs [32, 34]. Hsiao et al. also used MSNs as the templates to synthesize PLNPs which were loaded with afatinib (AFT) chemodrugs and attached with specific targeting aptamer (MAGE-A3) (Fig. 14a) [120]. Then they used these PLNPs for in situ inhabitation of lung adenocarcinoma progression. PersL imaging of orthotopic lung cancer models and isolated lung and H&E staining all confirm the therapeutic effect (Fig. 14b–d). Wang et al. firstly did the mesoporous silica-coated on the PNLPs, then extrude red blood cells membrane vesicles or Lactobacillus reuteri biofilm on the PLNPs@SiO_2_ surface to endow these nanocarriers with the ability to evade macrophage phagocytosis and systemic metabolism [70, 73]. Yan et al. constructed MSNs coated PersL nanoplatform (pHLIP-SS-GFLG-MSPLNPs @DOX), which had the properties of cathepsin B/glutathione dual-responsive drug release [121]. Own to the specific-response in the tumor microenvironment, the nanoplatform effectively released the DOX for cell killing and tumor inhibition. Liposomes, as the widely used nanocarriers, have the advantages of good biocompatibility and biodegradability. Thus Yan et al. employed liposome coated PLNPs (PLNPs-Liposome) for DOX loading and PersL imaging-guided chemotherapy, where these nanoformulations exhibited high DOX loading efficiency (69.2 ± 2.8%) and remarkable therapeutic capability for tumors [122]. In addition, Zeolitici imidazolate framework-8 (ZIF-8) has been commonly applied for pH-sensitive drug delivery due to the superior drug loading capacity and good biocompatibility [123]. Hence, PLNPs@ZIF-8 core–shell nanostructures were constructed for drug delivery, which achieved high DOX loading and tumor-specific drug release [124, 125].

**Table 3:** A summary of published works about PLNPs-based chemotherapy

PLNPs	Surface coating	Loaded drug	Loading capacity	Operated subject	Refs.
Hollow ZnGa_2_O_4_:Cr	BSA	DOX	181 mg/g	Animal (iv)	[54]
Mesoporous Zn_1.07_Ga_2.34_Si_0.98_O_6.56_:Cr_0.01_	BSA	DOX	62 wt %	Animal (iv)	[66]
Zn_1.1_Ga_1.8_Ge_0.1_O_4_:Cr^3+^	Mesoporous silica	DOX	4.5 wt%	cells	[32]
Gd_3_Ga_5_O_12_:Cr^3+^, Nd^3+^	Mesoporous Silica	DOX	8.5 wt%	Animal (it)	[34]
ZnGa_2_O_4_:Cr^3+^,Sn^4+^	Mesoporous Silica	Paclitaxel	187 mg/g	cells	[126]
ZnGa_2_O_4_:Cr^3+^,Sn^4+^	Mesoporous Silica	Afatinib	15 wt%	Animal (iv)	[120]
Zn_1.25_Ga_1.5_Ge_0.25_O_4_: Cr^3+^, Yb^3+^, Er^3+^	Mesoporous Silica, RBC vesicles	DOX	7.1 ± 0.3 wt%	Animal (iv)	[70]
Zn_1.25_Ga_1.5_Ge_0.25_O_4_: Cr^3+^, Yb^3+^, Er^3+^	Mesoporous Silica, Lactobacillus reuteri biofilm	Fluorouracil	8.5 wt%	Animal (it)	[73]
Zn_1.1_Ga_1.8_Ge_0.1_O_4_:Cr^3+^,Eu^3+^	Mesoporous silica	DOX	105.9 mg/g	Animal (iv)	[121]
Zn_1.1_Ga_1.8_Ge_0.1_O_4_:Cr^3+^	Liposome	Paclitaxel	69.2 ± 2.8 wt%	Animal (iv)	[122]
Zn_x_Ga_y_Ge_z_O_4_:Cr^3+^	ZIF-8	DOX	93.2 wt%	Animal (it)	[125]
ZnGa_2_O_4_:Cr^3+^	ZIF-8	DOX	90 wt%	Animal (iv)	[124]

### PLNPs based gene therapy

**Fig. 15:**
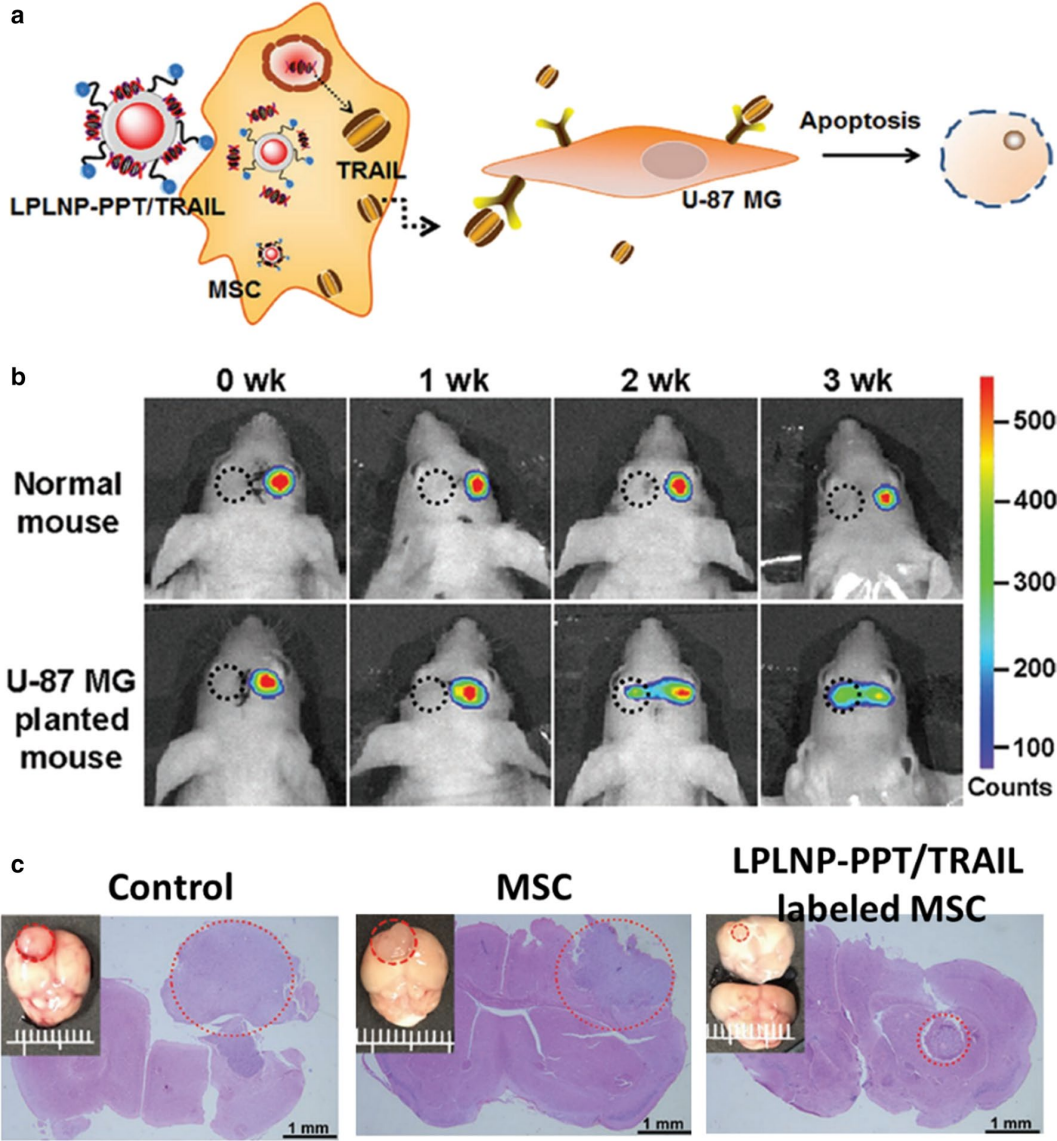
**a** A scheme for LPLNP-PPT/TRAIL based MSC tracking and gene therapy. **b** In vivo migration study of LPLNP-PPT labeled MSC. **c** Histology analysis of orthotopic brain tumor with different treatments. (Reproduced with permission [129]. Copyright 2020, Wiley–VCH)

Gene therapy has demonstrated high specificity, efficacy, and relatively few side effects in rehabilitation after surgical resection [127]. Han et al. designed a LED-responsive gene delivery system for localized gene therapy, where Gold nanorods and hTERT siRNA were col-loaded on ZnGa_2_O_4_:Cr^3+^ nanofibers. The gold nanorods absorbed the energy from LED radiated-ZnGa_2_O_4_:Cr^3+^ nanofibers to generate a mild photothermal effect and in turn induced the release of siRNA, which amplified the gene silencing effect [128]. Yan et al. constructed cell-penetrating TAT peptide and eGFP-TRAIL decorated PLNPs nanocomposite (PLNPs-PPT/TRAIL) for mesenchymal stem cells (MSC) tracking and effective therapy of glioblastoma (Fig. 15) [129]. The dual-functional nanocomposite not only enabled efficient targeting of MSC to induce therapeutic TRAIL ligand but also utilized afterglow to track the migration of MSC shifts over time.

### PLNPs based combined therapy

**Fig. 16:**
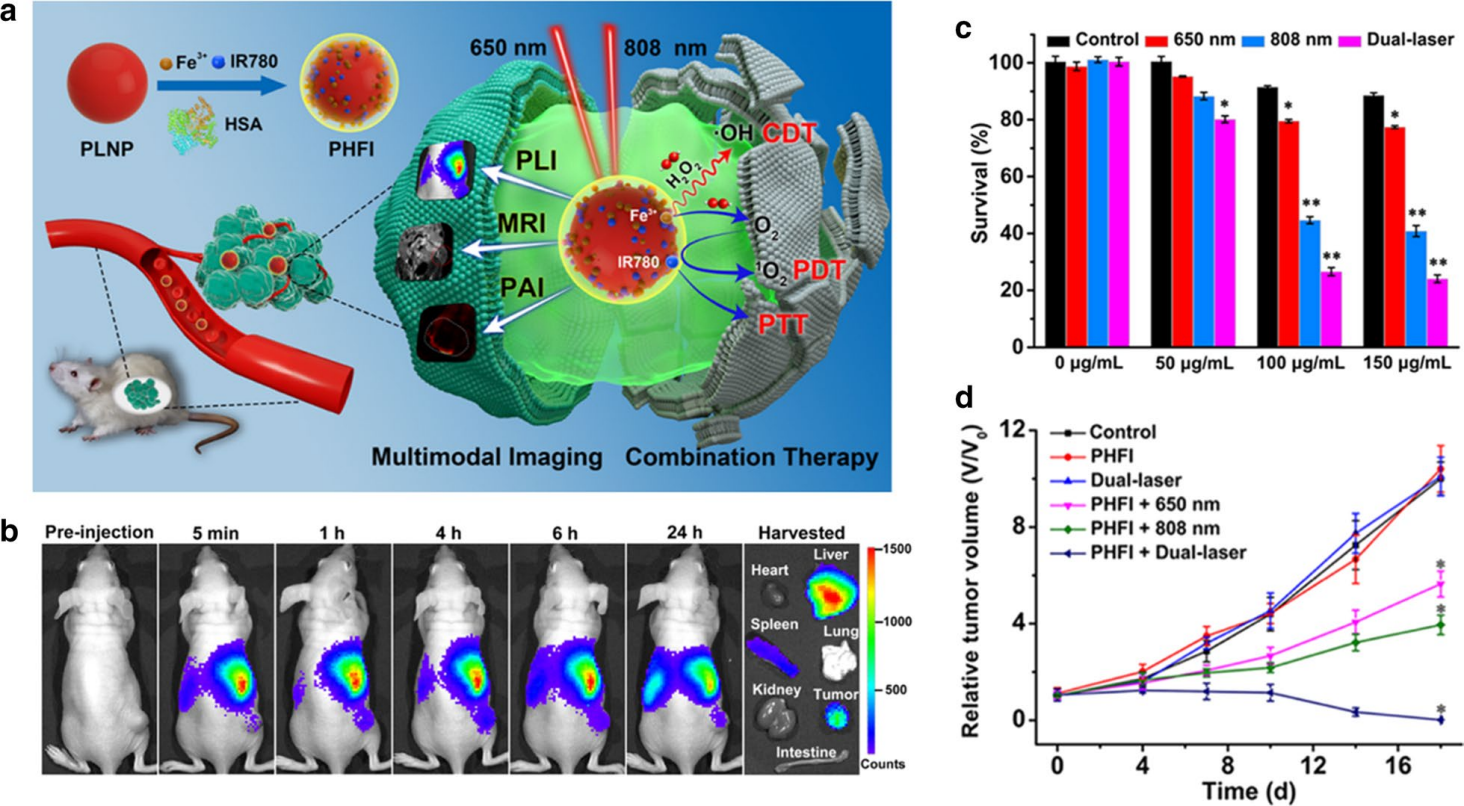
**a** Schematic representation of multimodal imaging and combination therapy. **b** In vivo PersL imaging of 4T1 tumor-bearing mice. **c** In vitro concentration-dependent cell viabilities of PHFI treated 4T1 cells with different laser irradiations. **d** Tumor-growth profiles of tumor-bearing mice with different treatment. (Reproduced with permission [130]. Copyright 2020, American Chemical Society)

Combined therapy can overcome the insufficient therapeutic effect of single therapy [9]. Therefore, Yan et al. developed a biomimetic PersL nanoplatform for metastasis tracking and chemophotodynamic therapy. The nanoplatform (DSPLNPs@hSiO_2_@CCM) were constructed on cancer cell membrane (CCM) and hollow silica multilayer coated PLNPs, which afforded the high loading capacity of Si–Pc and DOX in the nanoplatform. The reactivatable PersL from PLNPs not only provided long-term PersL imaging of metastases, but also was as an internal light source for Si–Pc activation, which enhanced the intracellular DOX release and achieved controllable combined chemophotodynamic therapy of metastases. Zhang et al. designed a PersL nanoplatform (PHFI) which co-doped human serum albumin (IR780 iodien and Fe^3+^) was coated on PLNPs (Fig. 16a) [130]. The PHFI were used for MR/PA/PersL imaging of tumors (Fig. 16b). Meanwhile, PHFI exhibited the Fenton-like chemodynamic therapy as well as phototherapy, which effectively achieved efficient tumor inhibition in vitro and in vivo (Fig. 16c,d). Wang et al. constructed cancer cell macrophage membrane-camoouflaged PLNPs-based nanoplatforms for combined PTT and chemotherapy of colorectal cancer [72]. The nanoplatforms were firstly coated PLNPs with mesoporous silica, then loaded with photothermal agent (IR825) and chemodrug (irinotecan), and lastly encapsulated into cell macrophage membrane. With the excellent tumor homologous adhesion and combined therapy effect, the colorectal tumors were obtained good inhibition. Sun et al. developed ^131^I labeled ZnPc(COOH)_4_ conjugated ZnGa_2_O_4_:Cr^3+^ nanoplatform (^131^I-ZGCs-ZnPcC4) for both radiation-induced PDT and radionuclide therapy (RT) [131]. ^131^I as the therapeutic radionuclides not only produced the gamma-ray for RT but also served as internal excitation source to activate ZnGa_2_O_4_:Cr^3+^ with long-lasting luminescence for further continuously generating PDT from ZnPc(COOH)_4_. Due to these self-activated therapies, ^131^I-ZGCs-ZnPcC4 could highly do good for deep tumor therapy. Recently, Yan et al. reported the pH-responsive cyanine conjugated PLNPs for PersL imaging of tumor and PTT/PDT combined therapy [132]. The conjugated cyanine offered the photothermal and photodynamic properties for tumor treatment.

## Conclusion and outlook

We summarized the current research progress of PLNPs in the synthesis, surface modification, and their applications in bioimaging and cancer therapy. Although great processes are made in the biomedical application of PLNPs, there remain some issues that deserve further studies.

1. Although many advances have been made in the synthesis of PLNPs, the morphological regulation of PLNPs still has some problems. With the development of PLNPs in biomedical applications, more advanced synthetic methods are needed to precisely control the morphology, particle size, surface properties, PersL intensity, and PersL time of PLNPs.
2. The excitation light source is an important factor affecting the biomedical application of PLNPs. Since the PersL time for pre-excitation by UV lamps is not sufficient to support the tumor accumulation of PLNPs, future studies will focus on NIR light, X-ray, and radionuclides as the light sources for PLNPs‘ excitation. Meanwhile, the emission of PLNPs in the NIR I or NIR II range can achieve better tissue transmittance. As PLNPs with NIR II emission have been seldom reported for bioimaging, thus, the development of PLNPs with excellent NIR II PersL will be one of the future research hotspots.
3. Although PLNPs can provide PersL with high sensitivity for disease diagnosis, it cannot provide all the information needed in the process of disease diagnosis and treatment. Therefore, PLNPs with multimodal imaging properties are paid attention for cancer diagnosis.
4. PLNPs as smart drug delivery systems can be used for PTT, PDT, chemotherpay, gene therapy, and combined therapy. The main challenge so far is how to construct PLNPs nanoplatform with high-effective loading, adequate protection of therapeutic payloads during circulation, target-specific delivery, sufficient cellular internalization.
5. As a new type of fluorescent nanomaterial, PLNPs are currently used in tumor diagnosis and treatment. Compared with other fluorescent nanoprobes, such as quantum dots and upconversion nanoparticles, their application scenarios in the biomedical field are relatively limited. In the future, more applications of PLNPs in the biomedical field will be developed, such as in vitro diagnostics, cell imaging, and antibacterial disinfection, etc.
6. The biosafety studies of PLNPs, as a novel bioimaging material, have also been the focus of attention of researchers. Although researchers have investigated the biotoxicity of PLNPs at multiple levels, including cellular and animal, and have achieved many research results, however, the biosafety studies of PLNPs are still in their infancy. In the future, more attention will be paid to deeper biotoxicity studies, such as the change of protein and gene at the molecular biology level, the chronic exposure toxicity, migration distribution, and transformation of PLNPs at the animal level.

## Data Availability

Not applicable.
